# In Silico to In Cerebro—Development of the Orexin Receptor Antagonist “Photorexant” for Photomodulation In Vitro and in Mouse Brains

**DOI:** 10.1002/anie.9938411

**Published:** 2026-07-30

**Authors:** Marcus Angermann, Marie H. Deventer, Vicente Ledesma‐Martin, Nuno Martinho, Alix Flake, Silvia Mori, Angel Nuñez, Jaime Pignatelli, Rita C. Guedes, Christophe P. Stove, Michael Decker

**Affiliations:** ^1^ Pharmazeutische und Medizinische Chemie Institut für Pharmazie und Lebensmittelchemie Julius‐Maximilians‐Universität (JMU) Würzburg Würzburg Germany; ^2^ Faculty of Pharmaceutical Sciences Department of Bioanalysis Laboratory of Toxicology Ghent University Ghent Belgium; ^3^ Research Institute for Medicines (iMed.ULisboa) Faculdade de Farmácia Universidade de Lisboa Lisboa Portugal; ^4^ Department of Anatomy Histology and Neurosciences Universidad Autónoma de Madrid Madrid Spain; ^5^ Department of Translational Neuroscience Cajal Research Center for Neuroscience Consejo Superior de Investigaciones Cientificas Alcalá de Henares Spain

**Keywords:** antagonist, computational chemistry, GPCR, neuromodulation, photopharmacology

## Abstract

In this study, we report the first photoswitchable small molecule dual ligands for the orexin receptor subtypes 1 (OX1R) and 2 (OX2R) as molecular tools for optical control of orexin signaling. The orexin system is critical for several physiological processes and has been increasingly implicated in the pathogenesis of various psychiatric disorders. We implemented a prospective structure‐based design approach, generating azobenzene‐containing derivatives of the FDA‐approved dual orexin receptor antagonist suvorexant with a computational workflow that featured a novel fragmentation and azobenzene‐fusion strategy. The derivatives were subsequently evaluated by molecular docking to ensure preservation of the characteristic binding mode of suvorexant. Top candidates were synthesized and characterized by photophysical and pharmacological methods, namely our recently developed β‐arrestin 2 and miniGα_q_ recruitment assays for both receptor subtypes. Optimization of the photoswitchable moiety yielded the highly potent antagonist “photorexant,” exhibiting an up to 11‐fold activity difference between photoisomers. A newly optimized assay protocol for the characterization of photoswitchable GPCR antagonists demonstrated dynamic, reversible photomodulation of orexin receptors by photorexant in vitro. Electrophysiological studies in hypothalamic and locus coeruleus neurons in mice demonstrated photomodulation of its antagonistic properties in vivo, positioning photorexant as an excellent tool for further investigation of orexin signaling.

## Graphical abstract

Following a structure‐based design approach, photoswitchable derivatives of the FDA‐approved drug suvorexant were designed, docked, synthesized, and characterized pharmacologically at the orexin 1 and 2 receptors (OX1R and OX2R). Optimized “photorexant” showed up to an 11‐fold difference in IC_50_ between photoisomers, enabled dynamic and reversible photomodulation in vitro, and successfully controlled locus coeruleus neuronal activity in mice.

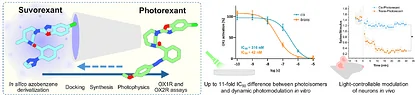

## 1 Introduction

The orexin (also termed hypocretin) system comprises two neuropeptides, orexin‐A (OXA) and orexin‐B (OXB), which are the endogenous agonists for the orexin 1 (OX1R) and orexin 2 (OX2R) receptors, two G protein‐coupled receptors (GPCRs) widely distributed across multiple brain regions [1, 2]. Its primary physiological functions include sleep–wake cycle regulation and modulation of feeding behavior [3, 4, 5]. Furthermore, orexin activity modulates neuronal excitability across multiple regions of the brain [6]. Orexin receptor antagonists have been developed for treating insomnia and obesity, with the dual orexin receptor antagonists (DORAs) suvorexant (Belsomra) [7], lemborexant (Dayvigo) [8], and daridorexant (Quviviq) [9] receiving FDA approval for the treatment of insomnia in 2014, 2020, and 2022, respectively. Meanwhile, agonists seem promising as effective treatments for narcolepsy but have yet to reach clinical approval [10, 11, 12, 13, 14]. Recent evidence also suggests that the orexin system plays a multifaceted role in the pathogenesis of neurodegenerative diseases such as Alzheimer's disease (AD), as several pathological hallmarks have been connected to changes in the orexin system [15, 16, 17, 18, 19]. Therefore, multiple studies and clinical trials are investigating the effectiveness of repurposing the FDA‐approved DORAs for the treatment of insomnia in AD specifically [11, 19, 20, 21, 22, 23, 24, 25, 26, 27].

Orexin neurons are a small population of neurons located at the lateral hypothalamus and perifornical region; however, they are broadly connected throughout the brain, participating in multiple physiological processes and behaviors. They do not only regulate sleep–wake cycles but are also involved in general energetic homeostasis and circadian rhythm inputs [28, 29, 30, 31, 32, 33, 34, 35]. Despite significant progress, the complexity of orexin connectivity and wiring remains elusive. Addressing these knowledge gaps requires the development of specialized chemical tools capable of probing orexin function with high spatiotemporal precision [36, 37].

Photopharmacology describes the concept of light‐activated drugs, whose pharmacological properties can be manipulated in a highly controlled manner [38, 39]. In 2022, Duffet et al. reported a photocaged OXB derivative that could be released upon UV light exposure, marking the first step toward optical control of orexin receptors [40]. Unlike photocages, which rely on an irreversible reaction, molecules incorporating photoswitchable moieties can be modulated in a reversible fashion, enabling more dynamic control through repeated cycles of activation and deactivation. Additionally, the insertion of a photoswitch into the original structure (a strategy known as “azologization” [41]) can help minimize changes to the parent compound and its pharmacological properties, rendering this approach ideal for exploring a wide range of physiological targets [42, 43, 44, 45, 46]. Reversible photomodulation of the orexin signaling system is currently underexplored. In a first approach, Prischich et al. recently reported a photoswitchable analog of OXB [47]. Peptides excel with regard to target selectivity, potency, and low toxicity, but can suffer from poor metabolic stability and rapid clearance [48, 49]. Small molecules, on the other hand, can be synthesized via simple, more versatile, and scalable synthetic procedures. Their vast design space also offers straightforward derivatization to finetune properties of the photoswitchable moiety, making them suitable for a broad range of settings and applications [50, 51, 52, 53].

Photoswitchable ligands are typically designed based on existing structural or structure–activity relationship (SAR) data to preserve the bioactivity of the parent molecule. Given the extensive information available regarding its pharmacodynamics and binding mode, we decided to investigate suvorexant as the template for our photoswitchable derivatives. Previous studies of orexin receptor–ligand structures have shown that the binding interactions of suvorexant and other antagonists are primarily hydrophobic, so we anticipated that the insertion of an azobenzene moiety into the parent structure would be well tolerated [54, 55]. Moreover, the well‐characterized binding mode and optimized structure of suvorexant (e.g., the chlorobenzoxazole moiety was found to significantly improve pharmacokinetic properties [56]) would provide a robust scaffold for ligand design and a promising starting point for potential in vivo evaluation of the newly developed compounds (Figure 1).

**FIGURE 1:**
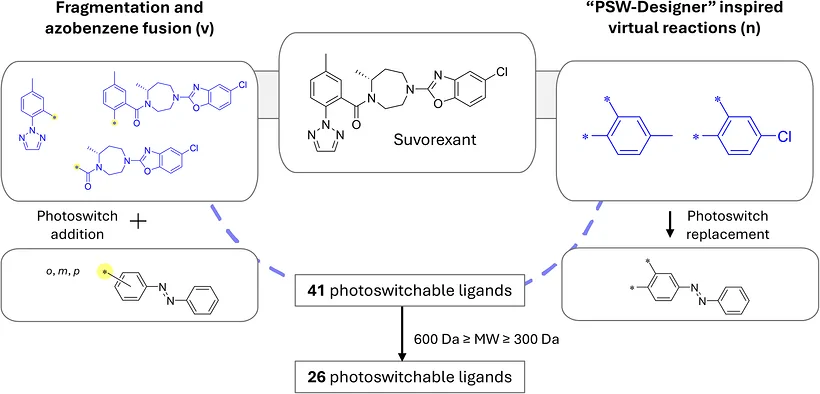
Computational workflow for the preparation of an azobenzene library derived from the structure of suvorexant. Fragmentation yielded 13 unique fragments, and the virtual reaction module identified two suitable moieties (methylphenyl and chlorophenyl) for azobenzene substitution. Azobenzene units were introduced at the *ortho*‐, *meta*‐, and *para*‐positions, resulting in 41 photoswitchable suvorexant derivatives, which were subsequently reduced to 26 candidates based on molecular weight (2D structures shown in Figure S54).

Recent studies have demonstrated that both structure‐ and ligand‐based virtual screening methods can successfully guide the discovery of photoswitchable compounds for multiple targets [57, 58, 59], allowing the exploration of a multitude of design hypotheses that may be otherwise overlooked while simultaneously steering away from those with a low probability of success. In our case, the availability of suvorexant‐bound orexin receptor structures enabled structure‐based virtual screening in two steps (Figure 1) [54, 60, 61, 62, 63, 64, 65]. First, a virtual library of photoswitchable derivatives was generated using “azologization” reactions inspired by the recently published “PSW‐designer” platform [59], expanded with a module employing fragmentation and azobenzene‐fusion to create photoswitches beyond predefined “azostere” substitutions. Second, the generated molecules were screened by molecular docking into the active sites of OX1R and OX2R to identify the most promising candidates. The docking results were assessed using interatomic distance calculations, comparing the predicted binding modes of the *cis*‐ and *trans*‐derivatives with suvorexant to ensure that the characteristic “horseshoe‐like” conformation, critical for receptor binding, was preserved [54]. This approach avoids overreliance on docking scores, which should generally not be directly taken as quantitative estimates of binding energies/affinities [57, 66, 67, 68, 69].

To ensure the accuracy and completeness of our computational findings, we conducted SARs of the azobenzene‐containing suvorexant derivatives and systematically investigated the influence of the position and attachment of the photoswitch on the pharmacological properties in our recently described cell‐based NanoBiT β‐arrestin 2 (βarr2) and miniGα_q_ recruitment assays [70]. Recent advances have highlighted the synergy between photopharmacology and computational chemistry [57, 59, 71, 72, 73, 74, 75, 76, 77]. Ligand‐based screening approaches have enabled the identification of photoswitchable ligands with light‐dependent activity at the retinoic acid receptor α and leukotriene‐A4 hydrolase [58]. Moreover, structure‐based virtual screening of photoswitches targeting GPCRs has shown potential, successfully identifying known photoswitchable ligands and their photoisomer‐specific activity in retrospective case studies for the β2‐adrenergic receptor and the histamine H3 receptor [59]. In this regard, to the best of our knowledge, this work represents the first prospective docking‐based virtual screening campaign aimed at designing novel photoswitchable GPCR ligands with subsequent systematic experimental validation.

Further optimization of the photoswitchable moiety solved key shortcomings, such as the switching wavelengths and poor photostationary distributions associated with the initial pharmacological screening set of molecules. Based on these results, a highly potent photoswitchable antagonist, coined “photorexant”, was synthesized and characterized in detail. We optimized the βarr2 recruitment assay protocol for the evaluation of photoswitchable GPCR antagonists to demonstrate reliable and dynamic in‐assay modulation of the compound's biological properties, achieving an effective switch of its antagonistic activity. Finally, successful photomodulation of its antagonistic properties was demonstrated through in vivo electrophysiology studies between hypothalamic and locus coeruleus (LC) neurons in anesthetized mice.

## 2 Results and Discussion

### 2.1 Virtual Azobenzene Library Generation and Screening Strategy

To guide rational design of photoswitchable suvorexant analogs, we developed a docking‐driven computational pipeline to evaluate a virtual library of previously unexplored, synthetically accessible suvorexant derivatives (Figure 1). Rather than serving as a *post hoc* evaluation, molecular docking was used as a design‐enabling filter constraining photoswitch incorporation to those substitutions that are compatible with the orexin receptor binding pocket. This structure‐aware approach is essential because introducing bulky azobenzene motifs can profoundly alter ligand geometry and binding orientation in ways that are not predictable by chemical intuition alone.

Built primarily using the open‐source cheminformatics Python package RDKit [78], our pipeline modifies the suvorexant scaffold through two complementary strategies: a fragmentation–fusion module and a reaction‐based azologization module inspired by the recently introduced “PSW‐Designer” platform [59]. In the fragmentation–fusion module (v), all non‐cyclic single bonds in suvorexant were systematically cleaved to generate molecular fragments. These were subsequently reassembled with an azobenzene motif installed at varying substituent positions (*ortho, meta, para*). In parallel, the reaction‐based module (n) applies a curated set of 31 virtual azologization reactions to identify substitution sites within the parent molecule. For suvorexant, this analysis highlighted the methylphenyl and chlorophenyl groups as viable sites for photoswitch replacement. By integrating both modules, we generated a library of 41 possible photoswitchable suvorexant derivatives, subsequently filtered by molecular weight (300–600 Da) to ensure retention of the core scaffold and the drug‐like physicochemical properties of small molecules (Figure S53). This exhaustive, unbiased strategy generates design hypotheses that would be impractical to enumerate experimentally.

The resulting library of 26 novel photoswitchable ligands was subsequently docked into the orthosteric binding sites of OX1R and OX2R using crystal structures co‐crystallized with suvorexant (PDB IDs: 6TO7 and 4S0V, respectively) (Figure S54, receptor preparation is detailed in the Supporting Information). The conserved water molecule mediating the hydrogen bond with H344/350^7 × 39^ (generic GPCRdb numbering [80], OX1R/OX2R residue) was retained, and energy minimization was carried out. During this process, the side chain of residue Q134^3 × 32^ in OX2R was automatically flipped to maximize the hydrogen bond network (Figure S55). To account for the potential impact of this conformational change on the docking results, an alternative OX2R structure was also included in which the orientation of Q134^3 × 32^ was reverted to its original crystallographic position.

Suvorexant adopts a highly characteristic “horseshoe‐like” conformation within the orexin receptor binding pocket, engaging predominantly in hydrophobic interactions, except for a direct hydrogen bond to N318/324^6 × 55^ and a water‐mediated hydrogen bond to H344/350^7 × 39^ (Figure S54). To ensure that the docked photoswitchable analogs preserved this binding mode, we calculated the root mean square deviation (RMSD) of the maximum common induced subgraph (MCIS) relative to the co‐crystallized suvorexant using the “rmsd2” algorithm implemented in the “fconv” command‐line tool [81] (results for OX1R shown in Figure 2A). Unlike standard RMSD calculations, this method identifies optimal atom mapping between non‐identical molecules, to calculate the displacement between shared substructures. Only azobenzene derivatives with an MCIS RMSD equal to or less than 2 Å compared to the co‐crystallized suvorexant in the corresponding orexin receptor structure (OX1R or OX2R) were retained for further analysis and filtered according to rational design criteria (Figure 2A). Compounds missing the central pharmacophoric amide (e.g., virtual compound **v14**) or having a carbonyl orientation that cannot form hydrogen bonds with N318/324^6.55^ (e.g., virtual compound **v20**) were discarded, even if they showed suvorexant‐like binding poses. Additionally, compounds featuring azo extensions to terphenyl‐like structures (e.g., virtual compounds **v11**, **v33**, and **v35**) were deprioritized due to expected solubility issues that would hinder biological testing.

**FIGURE 2:**
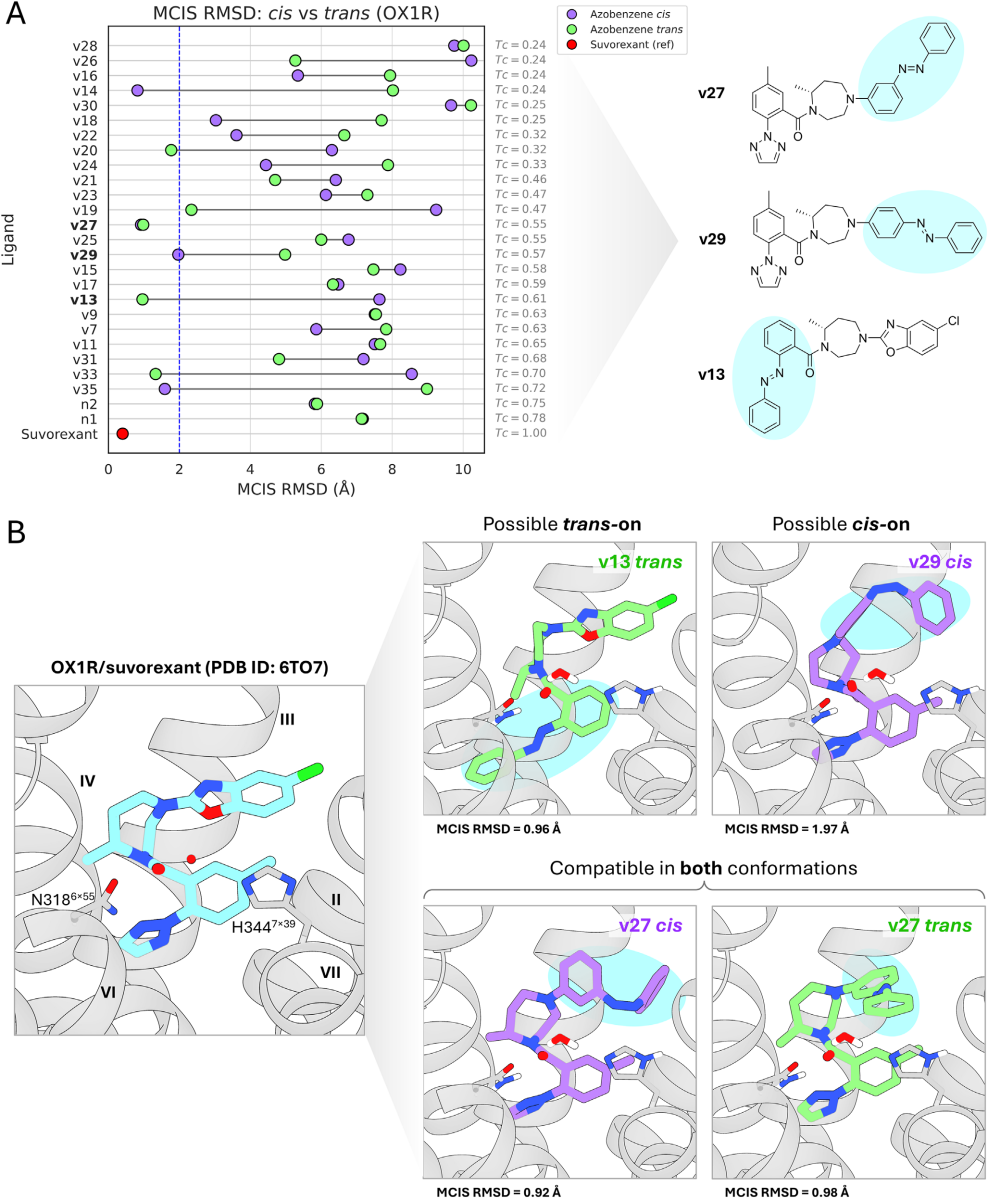
Docking results for virtual photoswitchable ligands at OX1R. (A) Root mean square deviation (RMSD) values of the docked ligands to the co‐crystallized suvorexant in OX1R (PDB ID: 6TO7), calculated over the maximum common induced subgraph (MCIS) atoms. The bottom‐most value corresponds to the self‐docking RMSD of suvorexant (docking validation, red sphere). Above it, MCIS RMSD values for the *cis*‐ and *trans*‐conformation of the photoswitches are shown, sorted by their 2D structural similarity (Tanimoto similarity over Morgan fingerprints, 2048 bits) to suvorexant (annotated on the right side of the plot as Tanimoto coefficient, *Tc*). The three selected hits and their 2D structures are shown on the right. (B) Predicted binding modes of the selected photoswitches compared to the experimental binding mode of suvorexant in OX1R. Transmembrane domains are labeled with Roman numerals. *Trans*‐photoisomers are colored green and *cis*‐photoisomers purple. Azobenzenes are highlighted in blue.

Our analysis of the OX1R results indicated that replacing either the chlorobenzoxazole or methylphenyltriazole fragments of suvorexant with an azobenzene moiety resulted in structures that would fit within the binding site while maintaining the overall binding mode (Figure 2B). Indeed, replacing the methylphenyltriazole with an *ortho*‐substituted azobenzene (virtual compound **v13**) was predicted to maintain the horseshoe binding mode in the *trans*‐conformation, with the azobenzene extension directed toward transmembrane helices 5 (TM5) and 6 (TM6). However, this binding mode was not predicted for the *cis*‐conformation, indicating a probable *trans*‐on antagonistic activity profile. In contrast, replacing the chlorobenzoxazole group with a *meta*‐substituted azobenzene (virtual compound **v27**) was compatible with both isomers, while the *para*‐substituted analog (virtual compound **v29**) was more consistent with a *cis*‐on antagonistic activity profile. Docking results for OX2R were different in terms of docking scores and poses (MCIS RMSD to suvorexant in PDB 4S0V, Figure S56). However, they did not yield additional hits beyond those identified for OX1R. Moreover, the orientation of residue Q134^3 × 32^ in OX2R (*vide supra*) did not substantially affect the docking results for most photoswitchable ligands. This suggests that the differences observed in the OX1R versus OX2R docking results are due to residue differences instead of side‐chain orientations. Specifically, residue S103^2 × 61^ in OX1R, which corresponds to T111^2 × 61^ in OX2R, and A127^3 × 33^ in OX1R, which is T135^3 × 33^ in OX2R, potentially influence ligand accommodation and receptor selectivity.

To validate the results of our virtual screening campaign, we followed up with an SAR approach. From virtual compound **v13**, a set of compounds was synthesized and characterized where the methylphenyltriazole amide of suvorexant was replaced with an *ortho*‐, *meta*‐, or *para*‐substituted azobenzene (compounds **1a–c**). We omitted the 7‐methyl substituent on the central heterocycle, which has been reported to result in a negligible loss of potency [63] (Scheme 1).

**SCHEME 1:**
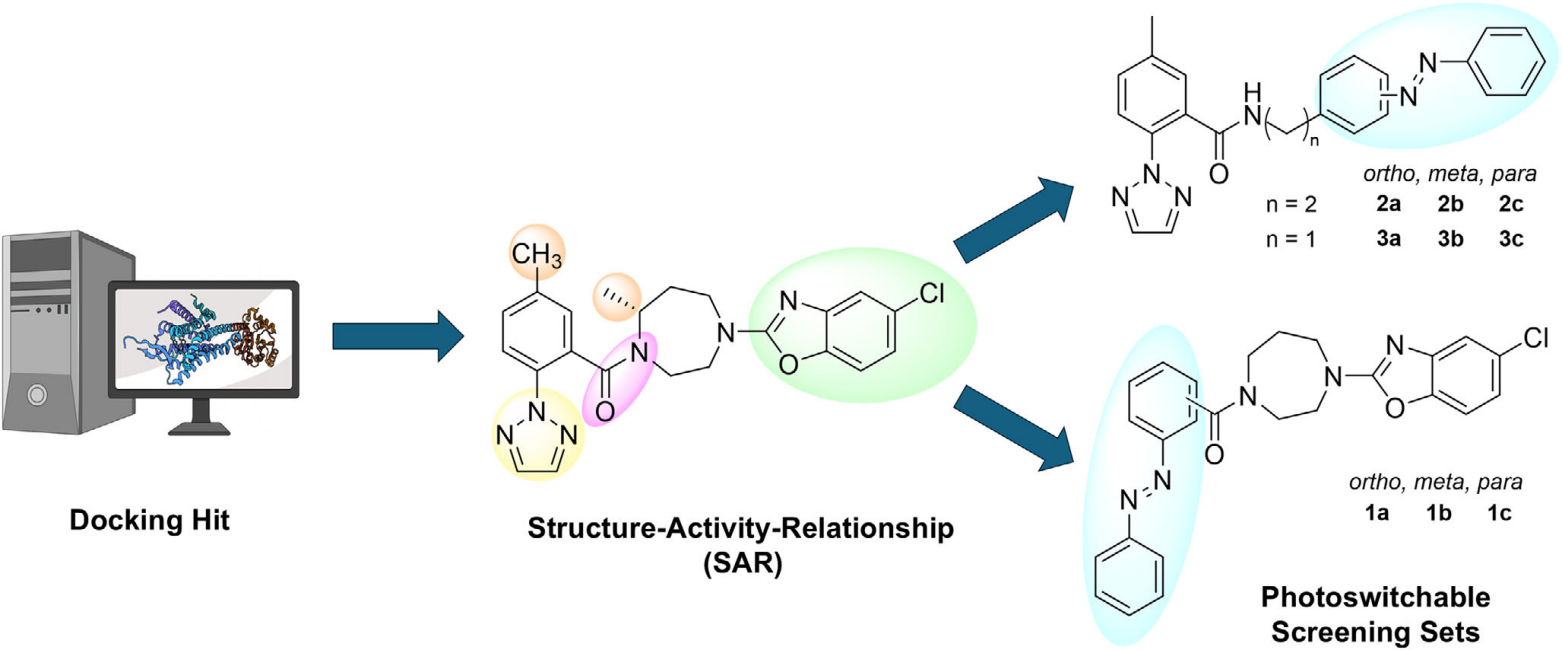
Comprehensive SAR investigation of azobenzene placement and attachment, aimed at experimentally validating the docking results, led to the design of three compound sets for pharmacological screening.

Conversely, virtual screening hits **v27** and **v29** featured the photoswitchable moiety on the opposite side of the ligand and binding pocket. Thus, we replaced the chlorobenzoxazole moiety with azobenzene. However, amino substitutions are known to result in short half‐lives, often in the range of seconds or less, which would not be feasible for subsequent cell‐based assays [82, 83, 84]. So, to circumvent this issue and facilitate synthesis, we replaced the central heterocycle with a simpler alkyl chain. We computationally evaluated whether the simplified alkylene linkers could adequately reproduce the spatial arrangement imposed by the diazepane ring in the virtual screening hits by ligand alignment (Figure S57, protocol detailed in the Supporting Information) and found alkylene linkers of length *n* = 1–3 suitable. Finally, we designed two screening sets, with *n* = 2 (compounds **2a–c**) and *n* = 1 (compounds **3a–c**) for the linker length, respectively (Scheme 1).

### 2.2 Synthesis

Synthesis of the amide‐based photoswitchable ligands **1a‐c** and **4** was achieved through convergent synthesis, starting with a single *Boc* protection of homopiperazine to compound **7** and substitution with 2,5‐dichlorobenzoxazole. This was followed by *Boc* deprotection to compound **9** and subsequent amide coupling to the respective photoswitchable ligands **1a–c** and **4** (Scheme 2).

**SCHEME 2:**
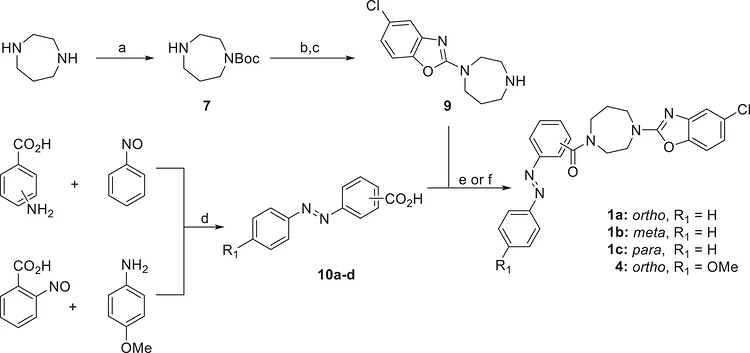
Synthesis of photoswitchable suvorexant derivatives **1a–c and 4**. (a) Boc_2_O, DCM, 0°C, 3 h, quant.; (b) 2,5‐dichlorobenzoxazole, Et_3_N, DMF, 90°C, 20 h, 66%; (c) TFA, DCM, rt, 5 h, 89%; (d) HOAc, rt, 18 h, 22%–84%; (e) HBTU, DIPEA, DMF, rt, 18 h, 85%–95%; (f) ethyl chloroformate, Et_3_N, DCM, rt, 18 h, 82%.

Synthesis of the alkylene‐linked photoswitchable derivatives **2a–c** and **3a–c** was achieved in two or three steps, respectively, starting with the Baeyer‐Mills reaction [85] of the corresponding aminobenzylamine to yield compounds **12a–c** or *Boc* protected 2‐aminoethylaniline to yield compounds **11a–c**, followed by deprotection with trifluoroacetic acid (TFA) and amide coupling with 5‐methyl‐2‐(2*H*‐1,2,3‐triazol‐2‐yl)benzoic acid (Scheme 3).

**SCHEME 3:**
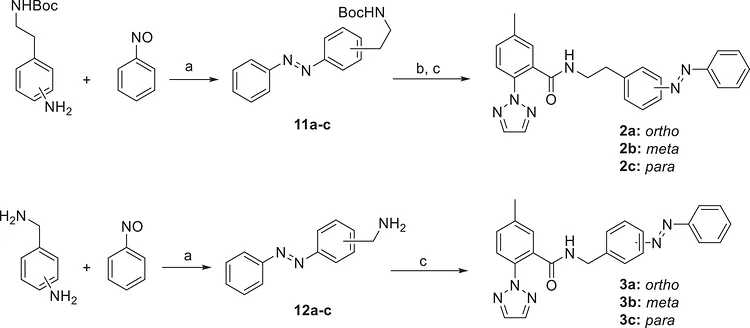
Synthesis of photoswitchable suvorexant ligands **2a–c** and **3a–c**. (a) HOAc, rt, 18 h, 28%–81%; (b) TFA, DCM, rt, 1 h; (c) HBTU, 5‐methyl‐2‐(*2H*‐1,2,3,­triazol‐2‐yl)benzoic acid, DIPEA, rt, 18 h, 68%–93%.

### 2.3 Photocharacterization

The photophysical properties of all synthesized target compounds were studied by UV‐Vis spectroscopy in a 25 µM solution containing dimethylsulfoxide (DMSO) and phosphate‐buffered saline (PBS) buffer at pH 7.4 [86, 87]. Photostationary states (PSS) were determined by liquid chromatography coupled to mass spectrometry (LC‐MS) (Table 1). All compounds could be reliably switched to the *cis*‐form with 365 nm light. However, some PSS values were unusually low for azobenzenes, such as the amide derivatives **1b–c** and the *meta*‐substituted photoswitches **1b**, **2b**, and **3b**. It is worth noting that the *cis*‐PSS of **1a** could later be improved from 61% to 95% by the introduction of an electron‐donating methoxy group in *para*‐position, shifting the absorption maximum from 327 nm to 360 nm in compound **4** (Table 1). Switching wavelengths, PSS values, stability against photofatigue, and thermal half‐lives were determined for all target compounds. A representative characterization of compound **1a** is shown in Figure 3. Full UV‐Vis and LC‐MS characterization data of all target compounds are available in the Supporting Information.

**TABLE 1:** Photophysical properties of photoswitchable suvorexant derivatives measured in a 25 µM solution of 1:1 PBS:DMSO.

Compound	*λ*_max_ (nm)^a^	PSS*_cis_* (%)^b^	PSS*_trans_* (%)^b^	*t*_1/2_ ^c^
**1a**	327	61	89	> 24 h
**1b**	329	42	86	> 24 h
**1c**	329	46	86	> 24 h
**2a**	331	75	87	> 24 h
**2b**	325	49	85	> 24 h
**2c**	330	76	81	> 24 h
**3a**	328	71	72	> 24 h
**3b**	330	38	85	> 24 h
**3c**	329	64	84	> 24 h
**4**	360	95	80	16 h

^a^ Absorption maxima of the *trans*‐photoisomer.

^b^ Photostationary states as determined by LC‐MS measurement.

^c^ Thermal half‐life determined by UV‐Vis spectroscopy at 23°C.

**FIGURE 3:**
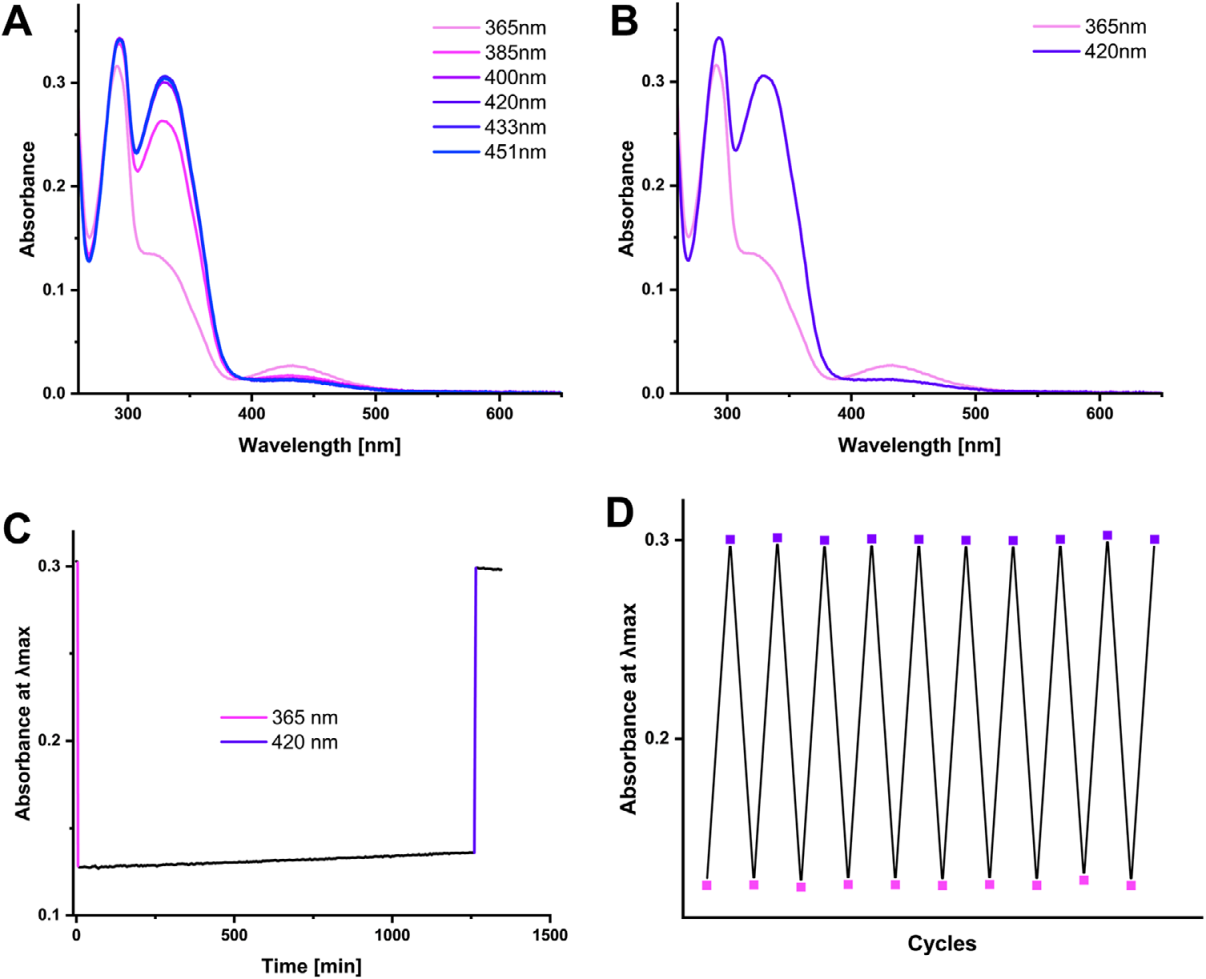
Representative photocharacterization of compound **1a** measured in a 25 µM solution of 1:1 PBS:DMSO at 23°C. (A) Absorption spectra recorded after irradiation with LEDs of different wavelengths. (B) Absorption spectra recorded after irradiation with the best *cis*‐ and *trans*‐photoisomerization wavelengths. (C) Thermal half‐life was determined by measuring the increase in absorbance after switching to the *cis*‐isomer. (D) Stability against photofatigue was confirmed by repeated irradiation with the *cis*‐ and *trans*‐photoisomerization wavelengths, respectively.

### 2.4 In Vitro Screening and Hit Identification

The synthesized compounds were first screened for antagonistic behavior at 10 µM and 100 nM in their *cis*‐configuration at both OX1R and OX2R, using βarr2 recruitment assays. A non‐photoswitchable reference, **5** [63] (a suvorexant‐derived analog lacking the 7‐methyl substituent, Supporting Information, page S53), was used as a positive control. The assay utilizes HEK293T cells expressing either OX1R or OX2R and employs the NanoBiT (Promega) principle. This is a functional complementation approach in which the orexin receptors are fused to LgBiT and βarr2 is fused to SmBiT, LgBiT and SmBiT corresponding to the large and small subunits of a split‐nanoluciferase, respectively. Luminescence was used as a readout for receptor activation and consequently antagonist efficacy. Following preliminary experiments, antagonism of the *cis‐* and *trans‐*isomers was assessed in the presence of 50 nM of the dual OX1R/OX2R agonist OXA. This concentration is high enough to activate the receptor, without being too high to be antagonized by potential moderate‐potency antagonists [70]. Compound **1c** could not be investigated due to solubility issues at the concentrations tested. Activity evaluation was primarily based on data obtained for the lower 100 nM concentration, as at 10 µM we could not exclude some interference (absorption) from the orange‐colored compounds with the emitted light from the luciferase enzyme [88, 89]. By screening the same set and concentrations at a different GPCR (the μ‐opioid receptor, MOR), we could confirm that the observed signal drop (below base level) seen for some compounds tested at 10 µM was a nonspecific effect, unrelated to orexin receptor binding and potentially caused by the color. For reference compound **5**, of which the stock solution was colorless, these effects were not observed (Figure S1). Two compounds, **1a** and **2b**, were identified and selected for further characterization based on their activity profiles at OX1R. The hits originated from two distinct screening sets and were consistent with the results of our computational studies, in which the *ortho‐*substituted virtual derivative **v13** and *meta*‐substituted virtual derivative **v27** had been identified as hits. Moreover, docking studies of *cis*‐**1a** produced a pose highly displaced with respect to suvorexant inside the binding site (MCIS RMSD of 6.12 Å in OX1R and 5.31 Å in OX2R with both Q134^3×32^ conformations), suggesting unlikely binding of this photoisomer at the receptor. Thus, the apparent antagonistic activity of *cis*‐**1a** may have been primarily caused by the residual *trans*‐photoisomer in the photostationary mixture (*vide supra*), a challenge that was later solved by further structural optimization. For both compounds, a concentration of 100 nM yielded a >40% reduction in OX1R activation by 50 nM OXA (Figure 4A). As at the OX2R only compound **1a** yielded a >50% reduction in response (Figure 4B), the decision to select compounds **1a** and **2b** for more in‐depth characterization (*vide infra*) was primarily based on the initial OX1R findings. Compounds from the third screening set (**3a–c**) showed minimal antagonistic activity, even at the highest concentration tested. Subsequent docking studies found similar poses for both compound **2b** and compound **3b** within the OX2R binding site (Figure S59), making it difficult to rationalize the difference in activity. Moreover, even though docking simulations suggested suvorexant‐like binding modes for the *para*‐substituted compounds from sets 2 and 3 (i.e., compound **2c** in *cis* and *trans* within OX2R, and compound **3c** in *cis* within both OX1R and OX2R, Figure S59), these molecules displayed the weakest antagonistic potency. This could point to a limitation of the docking protocol, which could be failing to capture the loss of favorable receptor–ligand interactions or the introduction of destabilizing contacts within the binding site. This could be better assessed through more exhaustive computational approaches such as molecular dynamics simulations and highlights the importance of experimental SAR validation of computational results [90].

**FIGURE 4:**
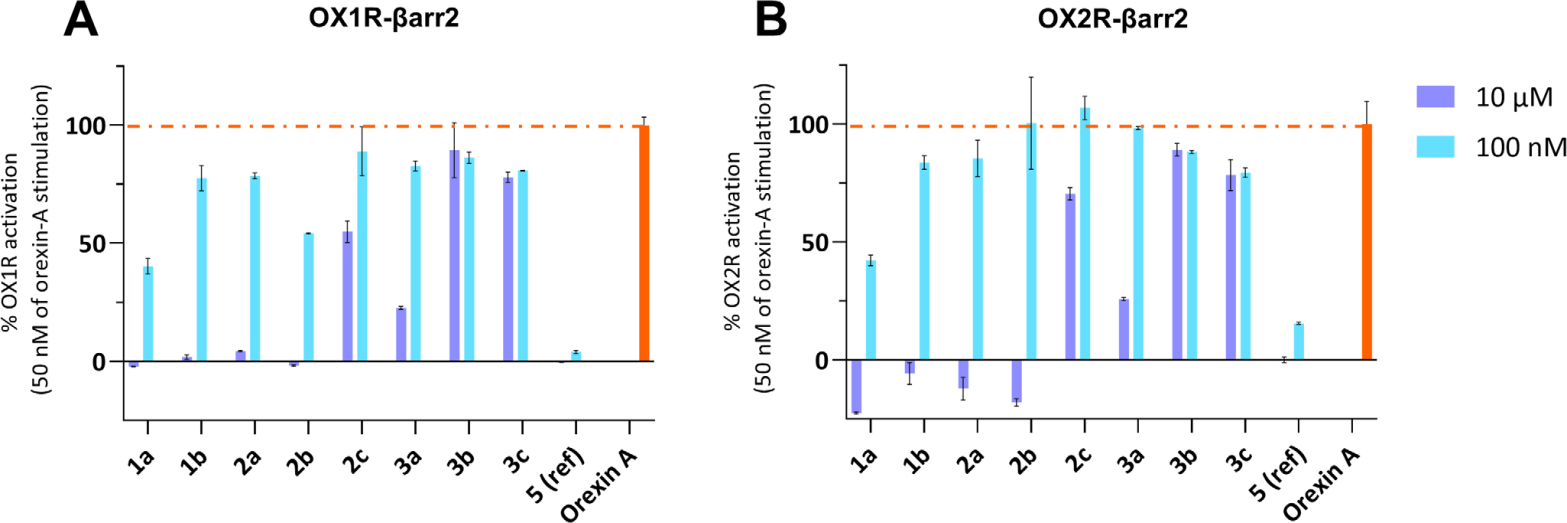
Bar charts showing antagonism in the OX1R‐βarr2 (A) and OX2R‐βarr2 (B) functional assays of 2 concentrations (10 µM and 100 nM) of 8 photoswitchable compounds, switched to the *cis*‐state, and the reference compound (**5**), in the presence of 50 nM of orexin‐A. Each bar represents the mean response ± SEM of at least *n* = 3 independent experiments, run in duplicate, and normalized to the effect of 50 nM of orexin‐A (orange, arbitrarily set as 100%).

### 2.5 Functional Evaluation of Photoswitchable Lead Compounds

The above‐mentioned βarr2 recruitment system was then utilized for further in‐depth evaluation of the activity of the selected photoswitchable antagonists **1a** and **2b**. Full concentration–inhibition curves were recorded (Figure 5). The corresponding derived IC_50_ values are shown in Table 2. It should be noted that for compounds **1a** and **2b** at 10 µM the apparent antagonistic effect may be overestimated due to nonspecific effects, as confirmed in the MOR control assay (cf. above, Figure S1). Overall, antagonists **1a** and **2b** showed a concentration‐dependent inhibition at the OX1R, blocking OXA from activating the receptor. Moreover, both compounds showed practically overlapping curves for both the *cis‐* and *trans‐*photoisomers, indicating only a small difference in antagonist behavior upon switching. In line with this, both isomers also displayed similar IC_50_ values. For compound **1a**, IC_50_ values of 241 nM (*cis*) and 143 nM (*trans*) were found, whereas for compound **2b** lower antagonistic potencies were observed (852 nM for the *cis‐*isomer and 386 nM for the *trans‐*isomer). Hence, in both instances, the *trans*‐isomers tended to have a higher antagonistic potency, as evidenced by the slightly lower IC_50_ values. Despite the expected reduction in inhibitory potential that is associated with an azologization approach, when compared to reference **5** (based on the rightward shift of the curve and the corresponding IC_50_ values), scaffold **1a** showed the most promising antagonist behavior, in agreement with the previous screening experiments. This reinforced the rationale for further optimizing this structure (*vide supra)*.

**FIGURE 5:**
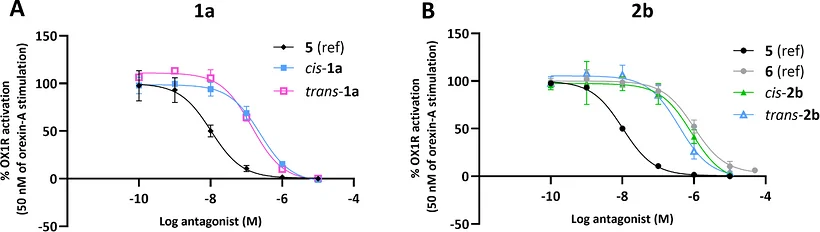
Concentration–inhibition curves of the *cis*‐ and *trans*‐isomers of photoswitchable antagonists **1a** (A) and **2b** (B) and their corresponding reference compounds at OX1R‐βarr2, in presence of 50 nM of orexin‐A. Data are normalized to the maximal effect of 50 nM of orexin‐A. Data are normalized to the *E*_max_ obtained for the blank‐treated condition. Each datapoint represents the mean ± standard error of the mean (SEM), obtained from a minimum of *n* = 3 independent experiments, run in duplicate.

**TABLE 2:** IC_50_ values of photoswitchable suvorexant derivatives at OX1R‐βarr2 and their respective 95% confidence intervals (CIs).

Compound	Photoisomer	IC_50_ (OX1R‐βarr2) [nM] (95% CI)	pIC_50_ (OX1R‐βarr2) (95% CI)	IC_50,_ *_cis_*/IC_50,_ *_trans_*
**1a**	*Cis*	241 (134–445)	6.62 (6.87–6.35)	1.7
	*Trans*	143 (94.0–220)	6.85 (7.03–6.66)	
**2b**	*Cis*	852 (420–1663)	6.07 (6.38–5.78)	2.2
	*Trans*	386 (237–623)	6.41 (6.63–6.21)	
**5** (ref)	—	10.3 (4.40–24.8)	7.99 (8.36–7.61)	—
**6** (ref)	—	994 (754–1307)	6.00 (6.12–5.88)	—

*Note*: The values were recorded in the presence of 50 nM orexin‐A and are the result of at least *n* = 3 experiments, run in duplicate.

Interestingly, while compound **2b** was less potent than reference **5** (from which its structure was initially derived), during our ongoing testing we found and subsequently synthesized an even closer structural non‐photoswitchable analog, previously described in literature and here referred to as reference compound **6** (Supporting Information, page S55) [91]. Both photoisomers of compound **2b** displayed a similar or even slightly higher inhibitory potential than the analogous non‐switchable scaffold (IC_50_ of 994 nM). This finding is particularly noteworthy, as the incorporation of a photoswitchable moiety is generally expected to drastically impair the pharmacological characteristics of compounds [43, 57]. In this way, we also report on a compound containing a photoswitchable group where the pharmacological behavior is conserved or even slightly improved when compared with the corresponding reference, which is quite rarely the case [92].

### 2.6 “Photorexant”—A Photoswitchable Dual Orexin Receptor Antagonist

Compound **1a** was chosen for further structural optimization due to a combination of higher antagonistic potency at 100 nM, superior PSS ratios, and closer structural similarity to the parent compound suvorexant. Previous studies showed that low PSS values may be caused by an unfavorable overlap between the absorption bands of the *cis*‐ and *trans*‐photoisomer [93, 94]. Introduction of electron‐donating substituents resolved this overlap by red‐shifting the π−π* transition of the *trans*‐photoisomer. We thus hypothesized that the addition of a methoxy group in the *para*‐position of azobenzene **1a** could improve PSS ratios for the same reason (*vide supra*). We confirmed that this modification would be tolerated through molecular docking (Figure 6), which indicates that the additional *para*‐methoxy group could be accommodated within the subpocket between TM5 and TM6 without significantly perturbing the overall binding mode of the *trans*‐photoisomer, while the *cis*‐photoisomer shows considerable displacement (Figure S61). Extension of ligands into this region was also supported by crystal structures of OX1R bound to the urea‐containing antagonists SB‐334867 (PDB ID: 6TQ7) and SB‐408124 (PDB ID: 6TQ9) (Figure S60) [54].

**FIGURE 6:**
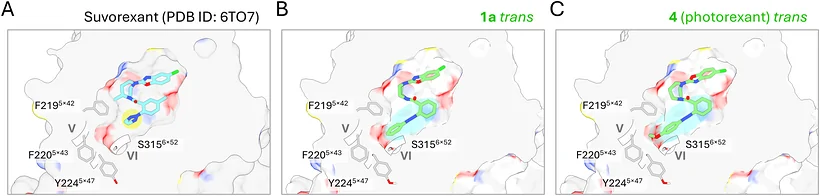
Subpocket between TM5 and TM6 in OX1R. (A) Crystal structure of suvorexant bound to OX1R (PDB ID: 6TO7), illustrating the methylphenyltriazole moiety (highlighted in yellow) occupying the TM5–TM6 subpocket. (B) Docking pose of *trans*‐**1a**. (C) Docking pose of the optimized *para*‐methoxy derivative *trans*‐photorexant (**4**), indicating that the azobenzene moiety and *para*‐methoxy substituent (both highlighted in blue) are accommodated within the same subpocket without altering the overall binding mode. Transmembrane domains are labeled with Roman numerals.

This led to the design and synthesis of compound **4**, later coined “**photorexant**.” It displayed both superior photophysical (Table 1) and biological (Figure 7 and Table 3) properties, providing quantitative switching to the less antagonistic *cis*‐photoisomer, with a pronounced difference between the IC_50_ values of the respective photoisomers, while restoring antagonistic potency to similar levels as the parent compound **5**.

**FIGURE 7:**
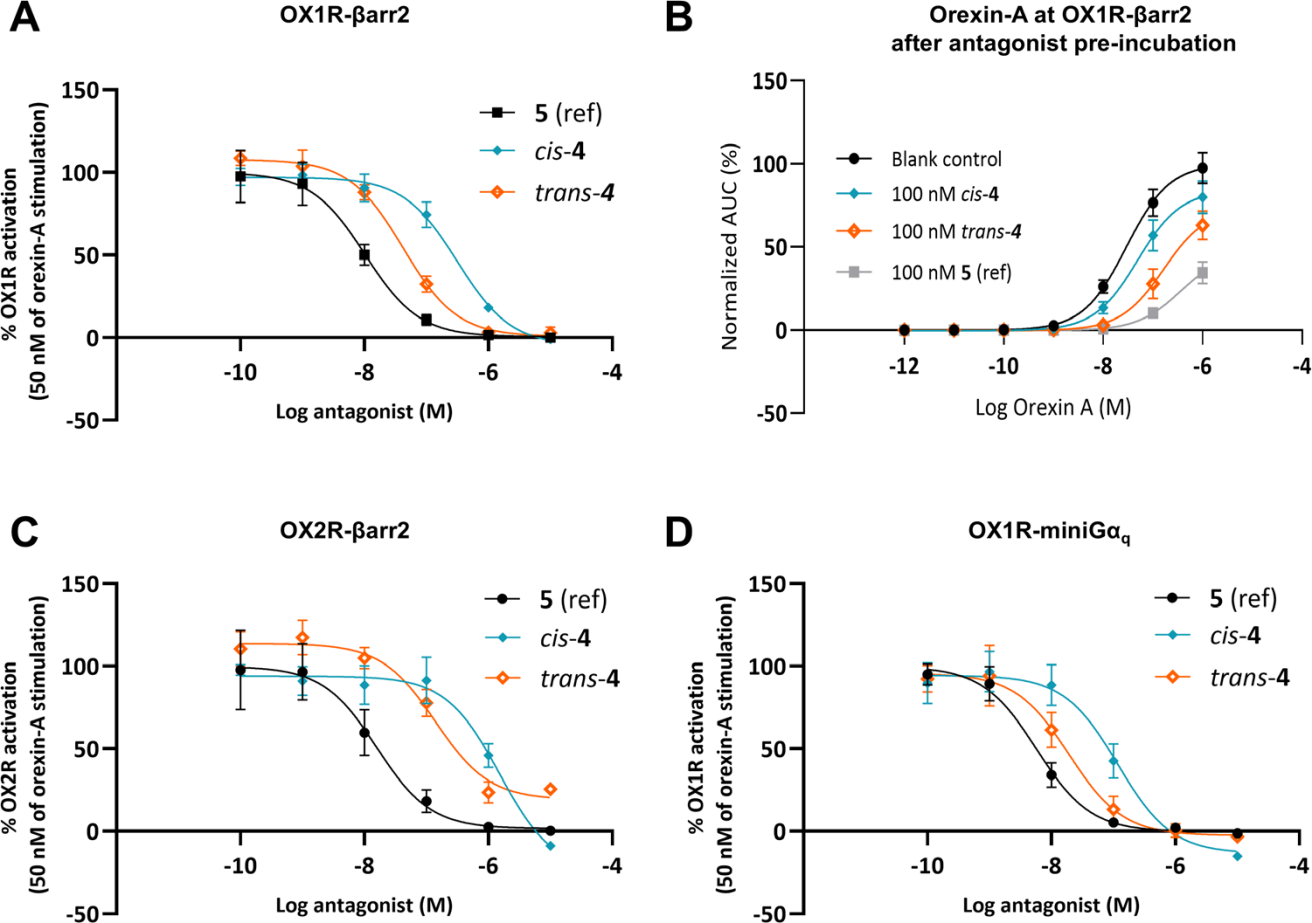
Concentration–inhibition curves of the *cis*‐ and *trans*‐isomers of photorexant (**4**) at OX1R‐βarr2 (panel A), OX2R‐βarr2 (panel C) and OX1R‐miniGα_q_ (panel D), in presence of 50 nM of orexin‐A. Panel (B) shows concentration–response curves of orexin‐A at OX1R‐βarr2 after pre‐incubation with either a blank solvent control, 100 nM of either *cis*‐**4** or *trans*‐**4**, or the non‐photoswitchable reference compound **5**. Data are normalized to the maximal effect of 50 nM of orexin‐A or the *E*_max_ of orexin‐A (panel B), and each data point represents the mean ± standard error of the mean (SEM), obtained from a minimum of *n* = 3 independent experiments, run in duplicate.

**TABLE 3:** IC_50_ values of photorexant (**4**) measured in various systems and their respective 95% confidence intervals (CIs).

Assay	Compound	Photoisomer	IC_50_ (nM) (95% CI)	pIC_50_ (nM) (pIC_50_ ± SEM) (95% CI)	IC_50,_ *_cis_*/IC_50,_ *_trans_*
OX1R‐βarr2	**4**	*Cis*	316 (174–574)	6.50 (6.76–6.24)	7.5
		*Trans*	42.2 (25.5–68.2)	7.38 (7.59–7.17)	
	**5** (ref)	—	10.3 (4.40–24.8)	7.99 (8.36–7.61)	—
OX2R‐βarr2	**4**	*Cis*	1522 (631–4362)	5.82 (6.20–5.36)	10.9
		*Trans*	140 (62.1–314)	6.85 (7.21–6.50)	
	**5** (ref)	—	15.8 (4.24–69.5)	7.80 (8.37–7.16)	—
OX1R‐miniGα_q_	**4**	*Cis*	118 (48.4–303)	6.93 (7.32–6.52)	6.1
		*Trans*	19.3 (7.47–54.1)	7.72 (8.13–7.27)	
	**5** (ref)	—	5.76 (3.18–10.3)	8.24 (8.50–7.99)	—

*Note*: The values were recorded in the presence of 50 nM o‐rexin‐A and are the result of at least *n* = 3 experiments, run in duplicate.

When tested in the βarr2 recruitment assay, the optimized photorexant (**4**) exhibited a stronger intrinsic antagonist potential in the *trans*‐photoform (IC_50_ = 42 nM) compared to its parent compound **1a** (IC_50_ = 143 nM). We tentatively attribute this to the improved filling of the subpocket between TM5 and TM6 (*vide supra*). Photorexant (**4**) displayed a clear difference between the *cis‐* and *trans*‐photoisomers (Figure 7A). Specifically, we found an over 7‐fold difference between the IC_50_ values, with the *trans*‐isomer (IC_50_ = 42 nM) being the more potent antagonist, compared to its *cis* counterpart (IC_50_ = 316 nM). We therefore report the successful synthesis of an orexin receptor antagonist whose pharmacological properties can be substantially altered via photomodulation.

To complement the generated concentration–inhibition curves for photorexant **4** at OX1R‐βarr2, we also performed the “inverse” experiment (Figure 7B). Rather than first incubating with a concentration range of photorexant, followed by adding a fixed concentration of OXA (50 nM, in‐well), cells were pre‐incubated with a fixed concentration of 100 nM of antagonist in the different states (*cis/trans*‐photorexant **4** or reference **5**) prior to running a concentration–response curve of OXA. In line with the above results, pre‐incubation with *cis‐* **4** only had a minor impact, while pre‐incubation with *trans*‐**4** clearly impeded receptor activation by OXA to a larger extent, as evidenced by a clear rightward shift of the OXA concentration–response curve. Next, we investigated whether the *cis/trans* difference was reproducible over different signaling pathways or receptor systems by assessing the antagonistic potential of *cis/trans* photorexant (**4**) in an assay recruiting the engineered miniGα_q_ protein to the OX1R and in an assay studying the recruitment of βarr2 to the OX2R. Our findings revealed that the differential behavior of the *cis‐* and *trans*‐photoisomers was consistent across the three assay systems, with the *trans*‐photoisomer displaying a 6‐ to 11‐fold higher antagonistic potency compared to the *cis*‐photoisomer (Figure 7C,D, Table 3). Thus, we demonstrated a robust modulation of its pharmacological properties, with only a limited loss of antagonistic potency when compared to reference compound **5**. Furthermore, we observed a slight shift toward an OX1R preference over OX2R. Note that the overall superior performance of photorexant compared to its parent compound **1a** is likely the result of a combination of multiple factors, such as improved intrinsic antagonist affinity of the *trans*‐photoisomer, improved PSS ratios and a lower antagonist affinity of the *cis*‐photoisomer; however, the exact contribution of each factor is not possible to quantify. Based on the concentration–inhibition curves, a concentration of 100 nM exhibited the most pronounced difference in antagonism between the two isomers, which is why we selected this concentration (as well as the slightly higher 1 µM) for further assessment of the (dynamic) photomodulatory behavior. Together, these results establish photorexant (**4**) as a promising photoswitchable antagonist for further investigations of the orexin system.

### 2.7 Photorexant Allows Dynamic Photomodulation

To investigate the reversibility of switching photorexant (**4**) after binding to OX1R (also known as dynamic photomodulation), we applied a setup similar to what has been described before by Gerwe et al. [95] and Sink et al. [96] for the investigation of photoswitchable agonists. As this requires irradiation during the assay, several preliminary experiments were conducted to ensure compatibility of the assay with irradiation and to obtain an optimized assay format. From control experiments (details can be found in the Supporting Information), we concluded that light of both 365 and 530 nm was well tolerated by the cells, the OXA peptide, and the nanoluciferase substrate—although a slight negative impact was observed for the latter. This indicates the suitability of both wavelengths for dynamic photomodulation experiments—keeping in mind the impact on the nanoluciferase substrate.

Test solutions were first brought into the *cis‐* or *trans‐*state and added to the OX1R‐βarr2 cells. After 30 min, the cells were irradiated for 3 min to convert the photoswitch to either the *trans*‐ or *cis*‐photoisomer, respectively, and this was repeated twice, alternating the irradiation wavelengths. In essence, we aimed to assess whether the antagonistic properties of photorexant (**4**) could be switched “on” (*trans‐*state) or “off” (*cis‐*state) while bound to the receptor. However, capturing differences in signaling behavior with an activation‐based assay is less straightforward for antagonists than for agonists. Once bound to the receptor, the antagonist could continue occupying the binding pocket to a certain extent, preventing receptor activation from the added agonist. In this regard, Binkhorst et al. recently employed a radioligand binding assay to investigate the in situ photomodulation kinetics of an H3 histamine receptor antagonist and found rapid loss of binding affinity upon switching, suggesting that prolonged blocking of the orthosteric pocket is not necessarily an issue. However, that study did not investigate functional receptor activation [97]. It is also important to note that due to the natural degradation of the substrate over time and a slight negative impact of the irradiation on the substrate, luminescence signals readily decreased “as such” while conducting the 2‐h assay. Optimization experiments revealed that the best way to cope with this issue was to add an additional “dose” of agonist (OXA, 10 or 50 nM in‐well concentration) after each irradiation step. This allowed us to assess whether irradiation and switching photorexant (**4**) to the *cis‐*photoisomeric state resulted in a loss of antagonistic behavior (and vice versa). As can be seen in Figure 8, in this scenario, OX1R activation following the addition of an agonist could not be counteracted by the *cis*‐state after irradiation at 365 nm, as scored by a clear increase in signal. In contrast, switching back to the *trans‐*state by irradiation at 530 nm yielded an effective antagonist, as scored by the absence of OX1R activation following additional OXA treatment. In comparison, irradiation of the non‐switchable reference **5** had no substantial impact on its antagonistic properties, as the additional administration of OXA did not result in receptor activation, irrespective of the irradiation.

**FIGURE 8:**
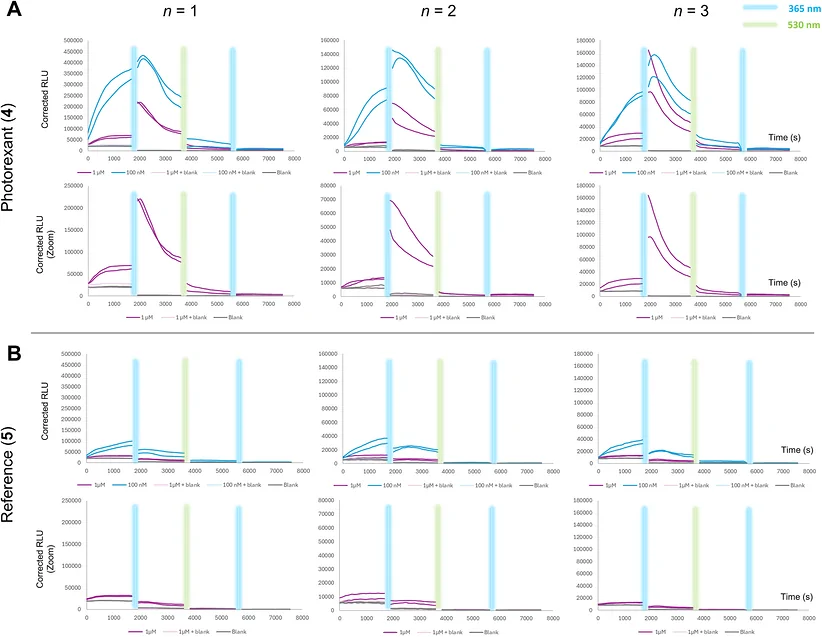
In vitro dynamic photomodulation of photorexant (**4**) measured in the OX1R‐βarr2 assay starting from the *trans*‐state with 10 nM orexin‐A added after each irradiation (panel A) and non‐photoswitchable control **5** (panel B). Three independent experiments (*n* = 1–3) run in duplicate are depicted. Top panels show the effects at 1 µM (purple lines) or 100 nM (blue lines) concentrations of the tested compounds. Bottom panels show a detailed view of the top panels at the 1 µM concentration. Compounds were irradiated each 30 min, starting from *trans*. The switching sequence of *trans*/*cis*/*trans*/*cis* is shown. Blue bars represent switching to *cis* with 365 nm, green bars represent switching to *trans* with 530 nm. After each irradiation, 10 nM in‐well concentration orexin‐A was added. RLU: relative light units.

Taken together, these data provide evidence of the promising in vitro reversibility of the behavior of photorexant (**4**). Furthermore, although complex, this newly established procedure and experimental setup now also allow for the characterization of other photoswitchable GPCR antagonists. Data for all performed photomodulation experiments can be found in the Supporting Information.

### 2.8 Photorexant Can Modulate Neuronal Projections In Vivo

To validate photorexant as a novel molecular tool in a physiological setup, we performed in vivo electrophysiological recordings in anesthetized mice. Neurotoxicity studies at HT22 cells had been assessed before and revealed no toxicity up to a concentration of 10 µM (details available in the Supporting Information, Figure S10). Orexin neurons innervate LC neuronal populations. Therefore, we electrically stimulated hypothalamic orexin neurons and recorded the electrophysiological activity in the LC for up to 30 min after injection of *cis*‐ or *trans*‐photorexant (Figure 9A,B). As expected, 10 min after *trans*‐photorexant administration, the number of spikes from LC neurons decreased significantly (spikes per stimulus before administration = 1.5, spikes per stimulus 10 min after administration = 0.3; *n* = 6) (Figure 9C). In contrast, administration of the *cis*‐irradiated compound failed to inhibit the LC activity (spikes per stimulus before administration = 1.3, spikes per stimulus 10 min after administration = 1.2; *n* = 6) (Figure 9C). The difference in LC electrophysiological response observed in the presence of *cis*‐ versus *trans*‐photorexant was statistically significant. Hypothalamic stimulation caused an evoked potential (EP) into the LC neurons, which was significantly reduced 10 min after photorexant administration compared to the baseline. (Figure 9D). Conversely, *cis*‐photorexant administration failed to inhibit hypothalamic stimulation of the LC as EP remained unaltered. These results highlight the potential of photorexant to be used for further in vivo studies of orexin signaling.

**FIGURE 9:**
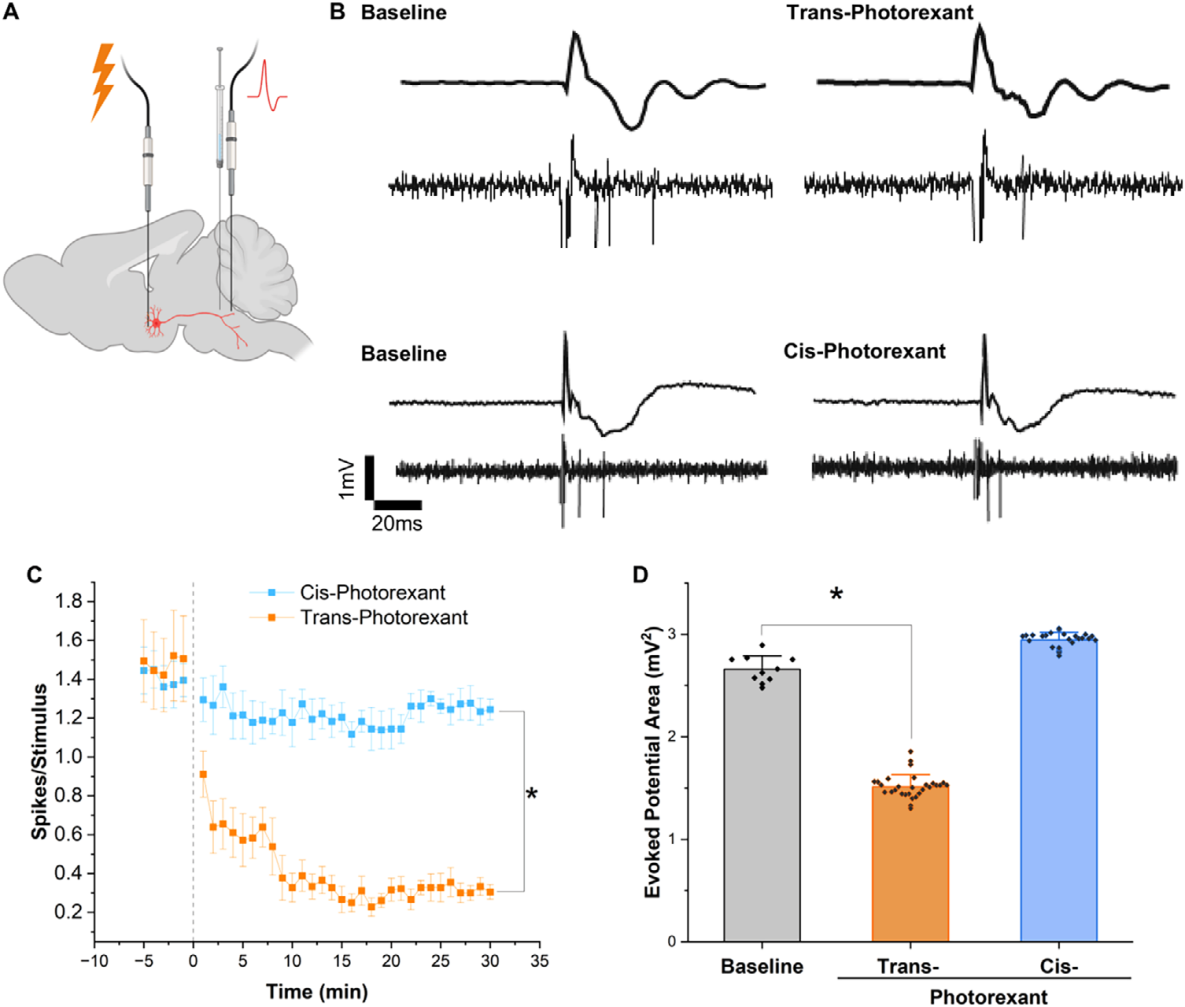
In vivo electrophysiological effects of *cis*‐ and *trans*‐photorexant (**4**) on locus coeruleus (LC) neurons. (A) Experimental setup for electrophysiology experiments. (B) Stimulation of hypothalamic neurons caused a measurable difference in LC neuronal activity (baseline). The orexinergic origin of this input can be modulated by photorexant (**4**) in its *trans* isomer (*trans*‐photorexant) but not in its *cis* isomer (*cis*‐photorexant). (C) Recorded spikes per stimulus before and after addition of *cis*/*trans*‐photorexant (**4**), *n* = 6, (ANOVA multiple repeated measures; *trans*‐photorexant = 0.5560 ± 0.069; *cis*‐photorexant = 1.2466 ± 0.01435; *F*‐value (*trans* vs. *cis*) = 492.290; *p* < 0.0001). Values represent the mean between 0 and 30 min after administration. (D) Evoked potential (EP) in the LC neurons after hypothalamic stimulation (*n* = 6). *Trans*‐photorexant baseline = 2.551 ± 0.0256; *trans*‐photorexant = 1.511 ± 0.022; one‐way ANOVA. *F*‐value (baseline vs. *trans*) = 1466.41672; *p* < 0.0001. *Cis*‐photorexant baseline = 2.767 ± 0.0369; *cis*‐photorexant = 2.945 ± 0.0137.

## 3 Conclusion

Herein, we report the first photoswitchable small molecule ligands for the orexin 1 and 2 receptors. They were developed by combining a computational design approach of the FDA‐approved dual orexin antagonist suvorexant with experimental structure‐activity relationships. We were able to identify two separate azobenzene‐containing scaffolds that allow spatiotemporal control of their pharmacological properties. The key modification, introduction of a methoxy group to the *ortho*‐amide photoswitch, was supported by docking experiments and resulted in the discovery of “photorexant,” with both superior photophysical and biological properties. It features quantitative switching to the *cis*‐photoisomer and a pronounced 6‐ to 11‐fold difference in IC_50_ values between the two photoisomers, with the *trans*‐photoisomer displaying the strongest antagonistic potential across three different assay systems (OX1R‐βarr2, OX2R‐βarr2, and OX1R‐miniGα_q_), while retaining most of the potency of its parent compound. To the best of our knowledge, this work represents the first prospective structure‐based virtual screening campaign of newly designed photoswitchable GPCR ligands with subsequent systematic experimental validation. A certain limitation of this study is the lack of off‐target screening, which can only be done for the dark‐adapted photoisomeric form in a commercial setting, and was therefore not performed. Since we have designed the compound based on suvorexant as outlined above, we do not expect such affinities, but further applications still might consider this.

Furthermore, utilizing a newly optimized assay protocol, which now allows recording dynamic signals of photoswitchable GPCR antagonists, we demonstrated reversible modulation of photorexant in cell‐based studies and were able to conduct successful photomodulation in vitro. Finally, electrophysiological experiments in mice demonstrated the ability of *trans*‐, but not *cis*‐photorexant, to inhibit the stimulation of the LC by the orexin neurons from the hypothalamus in vivo, further strengthening the potential of this compound to be used as a molecular tool in the elucidation of the complex signaling network of the orexin system and its implication in AD and other neurodegenerative disorders.

## Author Contributions

**Marcus Angermann**: conceptualization, investigation, validation, visualization, formal analysis, writing – original draft, data curation. **Marie H. Deventer**: investigation, validation, methodology, formal analysis, visualization, writing – original draft, data curation. **Vicente Ledesma‐Martin**: investigation, methodology, validation, formal analysis, writing – review and editing, data curation, writing – original draft, software. **Nuno Martinho**: methodology, formal analysis. **Alix Flake**: investigation, formal analysis, data curation. **Silvia Mori**: investigation, data curation. **Angel Nuñez**: formal analysis, data curation, investigation, methodology. **Jaime Pignatelli**: investigation, validation, methodology, writing – review and editing, conceptualization, visualization, formal analysis, project administration, data curation. **Rita C. Guedes**: conceptualization, project administration, funding acquisition, writing – review and editing, supervision. **Christophe P. Stove**: conceptualization, funding acquisition, methodology, project administration, writing – review and editing, supervision. **Michael Decker**: conceptualization, funding acquisition, writing – review and editing, project administration, supervision, resources, writing – original draft, methodology.

## Conflicts of Interest

The authors declare no conflicts of interest.

## Supporting information



The authors have cited additional references within the Supporting Information [78, 98, 99, 100, 101, 102, 103, 104, 105, 106, 107].
**Supporting File**: anie73670‐sup‐0001‐SuppMat.docx.

## Data Availability

The data that supports the findings of this study are available in the supplementary material of this article.
