# Supplementary material for: In Silico to In Cerebro—Development of the Orexin Receptor Antagonist “Photorexant” for Photomodulation In Vitro and in Mouse Brains
Source: Angew Chem Int Ed Engl. 2026 Jul 30;65(42):e9938411. doi: 10.1002/anie.9938411 (PMC13653633; doi:10.1002/anie.9938411)
Supplement: Supplementary file 1 [file ANIE-65-e9938411-s001.docx]

Supporting information

*In Silico to in Cerebro—Development of the Orexin Receptor Antagonist “Photorexant” for Photomodulation in Vitro and in Mouse Brains*

Marcus Angermann^+[a]^, Dr. Marie H. Deventer^+[b]^, Vicente Ledesma-Martin^+[a,c]^, Dr. Nuno Martinho^[c]^, Alix Flake^[a]^, Silvia Mori^[a,c]^, Prof. Dr. Angel Nuñez^[d]^, Dr. Jaime Pignatelli^[e]^, Prof. Dr. Rita C. Guedes^[c]^, Prof. Dr. Christophe P. Stove^[b]^, Prof. Dr. Michael Decker^*[a]^

[a] M. Angermann^+^, A. Flake, S. Mori, Prof. Dr. M. Decker
Pharmazeutische und Medizinische Chemie
Institut für Pharmazie und Lebensmittelchemie, Julius-Maximilians-Universität (JMU) Würzburg
Am Hubland, 97074 Würzburg, Germany
E-mail: michael.decker@uni-wuerzburg.de

[b] Dr. M. H. Deventer^+^, Prof. Dr. C. P. Stove
Laboratory of Toxicology, Department of Bioanalysis
Faculty of Pharmaceutical Sciences, Ghent University
Ottergemsesteenweg 460, 9000 Ghent, Belgium

[c] V. Ledesma-Martin^+^, Dr. N. Martinho, Prof. Dr. R. C. Guedes
Research Institute for Medicines (iMed.ULisboa)
Faculdade de Farmácia, Universidade de Lisboa
Av. Prof. Gama Pinto, 1649-003 Lisboa, Portugal

[d] Prof. Dr. A. Nuñez
Department of Anatomy, Histology and Neurosciences
Universidad Autónoma de Madrid
28029 Madrid, Spain

[e] Dr. J. Pignatelli
Department of Translational Neuroscience
Cajal Research Center for Neuroscience, Consejo Superior de Investigaciones Científicas
1, De León Avenue, 28805 Alcalá de Henares, Madrid, Spain.

+ These authors contributed equally.

**Table of Contents**

[**Biological Experiments**	3](#_Toc219468855)

[**Chemicals and Reagents**	3](#_Toc219468856)

[***In Vitro* Activity-Based Bioassays**	3](#_Toc219468857)

[**Dynamic Photomodulation of Photorexant**	13](#_Toc219468858)

[**Neurotoxicity Characterization of Photorexant**	17](#_Toc219468859)

[***In Vivo* Characterization of Photorexant**	18](#_Toc219468860)

[**Photochemistry**	19](#_Toc219468861)

[**General Methods**	19](#_Toc219468862)

[**Photocharacterization Data**	20](#_Toc219468863)

[**Determination of PSS Ratios**	30](#_Toc219468864)

[**Synthetic Procedures**	40](#_Toc219468865)

[**General Methods**	40](#_Toc219468866)

[**Synthetic Procedures**	41](#_Toc219468867)

[**NMR and LC-MS Characterization**	56](#_Toc219468868)

[**LC-MS Data of Target Compounds**	56](#_Toc219468869)

[**NMR Spectra**	68](#_Toc219468870)

[**Computational Methods and Results**	107](#_Toc219468871)

[**Docking Protocol**	107](#_Toc219468872)

[**Alkyl Linker Length Analysis**	107](#_Toc219468873)

[**References**	113](#_Toc219468874)

# **Biological Experiments**

## **Chemicals and Reagents**

For cell culture, Dulbecco’s modified Eagle’s medium (DMEM) (GlutaMAX^TM^), Opti-MEM I Reduced Serum, trypsin-EDTA (0.05%) penicillin, streptomycin and amphotericin B were purchased from Thermo Fisher Scientific (Waltham, MA, United States). Poly-D-lysine and fetal bovine serum (FBS) were from Sigma-Aldrich (Darmstadt, Germany). The FuGENE^©^ HD transfection reagent, Nano-Glo® Live Cell reagent and the Nano-Glo® LCS Dilution buffer were obtained from Promega (Madison, WI, United States). Methanol (MeOH) was procured from Chem-Lab NV (Zedelgem, Belgium), and dimethylsulfoxide (DMSO) was purchased from Sigma-Aldrich. The orexin A (human) reference standard was purchased from Bachem (Bubendorf, Switzerland).

## ***In Vitro* Activity-Based Bioassays**

Pharmacological characterization at OX1R and OX2R of the compounds described in this study was performed using *in vitro* live cell activity-based assays.

The principle of the assays is based on the NanoLuc® Binary Technology (NanoBiT®) by Promega and relies on the functional complementation of a split nanoluciferase enzyme. In essence, two inactive subunits of the luciferase enzyme, Large BiT (LgBiT) and Small BiT (SmBiT), are fused to the protein interaction partners of interest. One subunit (here LgBiT) is linked to the OX1R or OX2R, whereas the other subunit (SmBiT) is fused to the intracellular signaling partner, β-arrestin 2 (βarr2) or the engineered miniGα_q_. Following receptor activation by an agonist, the intracellular signaling protein is recruited, resulting in the 2 subunits coming into proximity. This event leads to functional complementation of the enzyme and restoration of the luciferase enzymatic activity. In presence of the substrate (furimazine), bioluminescence is generated as a measure of receptor activation.

**Transfection of HEK293 Cells**

For the different assays, the following plasmids were used: OX1R-LgBiT, OX2R-LgBiT, SmBiT-βarr2 and SmBiT-miniGα_q_. The generation of the plasmids and the assay setup has been described previously^[1–4]^. In short, human embryonic kidney cells (HEK293T) served as the vehicle for the assays and were routinely cultured in a humidified atmosphere at 37°C with 5% CO_2_ in DMEM (GlutaMAX^TM^) supplemented with 10% heat-inactivated FBS, 100 IU/mL of penicillin, 100 µg/mL of streptomycin, and 0.25 µg/mL of amphotericin B. The assays were performed in a transient format following a four-day protocol. On the first day, HEK293T cells were seeded in 6-well plates at 500,000 cells/well and incubated overnight. The next day, cells were transiently transfected using FuGENE® HD transfection reagent according to the manufacturer’s protocol (ratio of FuGENE:DNA 3:1). Each well was transfected with 1.65 µg of a plasmid containing the receptor (either OX1R-LgBiT or OX2R-LgBiT) and 1.65 µg of a plasmid containing the SmBiT-linked intracellular signaling protein (either βarr2 or miniGα_q_). On day three, transfected cells were reseeded on opaque poly-D-lysine-coated 96-well plates at 50,000 cells/well and incubated overnight.

**Orexin Assays**

On the final day, cells were rinsed twice with 150 µL of Opti-MEM I Reduced serum medium, and 100 µL was then added to the wells as the assay buffer. The Nano-Glo® Live Cell reagent, containing the furimazine substrate, was diluted 20-fold in Nano-Glo® LCS Dilution buffer, according to the manufacturer’s protocol. Subsequently, 25 µL of this substrate solution was added to the wells and the plate was placed in a TriStar^2^ LB 942 Multimode Microplate Reader (Berthold Technologies GmbH & Co., Germany). Luminescence was recorded during an initial equilibration period, with these data being used to correct for inter-well variability^[2]^.

Stock solutions of the target and reference compounds were made and used within 24 h in the assay and kept at 4°C. Compounds were diluted in DMSO and stock solutions were prepared via serial dilution in Opti-MEM. Appropriate solvent controls were included in all experiments.

**Pharmacological Screening**

For screening experiments in the OX1R-βarr2 and OX2R-βarr2 assays, compounds were tested at 10 µM and 100 nM (in-well concentration) and were switched to the *cis*-state by irradiation for at least 10 min at 365 nm, shielded from additional light sources. To obtain the *trans*-state, photoswitchable compounds were incubated in the dark overnight, at 37 °C (for compound **4**, additional testing was done to ensure this condition did not impact the assay, as detailed further and in **Figure S4**). Then, 10 µL of the test compounds were added to the wells for an incubation of approximately 5 min to let the (potential) antagonists occupy the receptor. After this, 10 µL of orexin-A (50 nM, in-well concentration) was administered and the plate was placed back into the luminometer for the final 2 h measurements. For screening at the µ-opioid receptor (MOR), a MOR-βarr2 recruitment assay was used, as previously described^[5]^. Cells were pretreated for 5 min with 10 µM or 100 nM of test compounds. Subsequently, either 10 µM hydromorphone or solvent control were added and luminescence was monitored for 2 h.

**Concentration–Inhibition Curves at the Different Orexin Receptor Assay Systems**

Concentration–inhibition curves were generated using three receptor–transducer systems:OX1R-βarr2, OX2R-βarr2, and OX1R-miniGα_q_. Similar to the screening, cells were pre-incubated with a concentration range of the test antagonists (either in the *cis*- or *trans*-state) and corresponding reference standards for approximately 5 min. Subsequently, 10 µL of orexin-A (50 nM, in-well concentration) were added and luminescence was monitored during 2 h. Additionally, OX1R-βarr2 cells were treated with 100 nM of compound **4** (either in the *cis*- or trans-state), the reference compound **5**, or solvent control, and left to incubate for approximately 5 min. Then, a concentration gradient of orexin-A was added and luminescence was recorded for 2 h.

**Data Analysis**

Absolute luminescence values were corrected in Microsoft Excel 2024 for inter-well variability using data obtained during the initial equilibration phase^[2]^. Area under the curve (AUC) values were calculated for all test conditions and blank-corrected by subtracting the AUC values of the corresponding blanks. For initial screening, data were normalized by arbitrarily setting the AUC of 50 nM of orexin-A to 100%. For subsequent pharmacological characterization, data were also normalized to the maximal effect of 50 nM of orexin-A. Orexin-A concentration–response curves (See **Figure 5B**) were normalized to orexin-A (after pre-incubation with solvent control). GraphPad Prism version 10.5.0 (San Diego, CA, USA) was used to generate concentration-inhibition or concentration–response curves and calculate IC_50_ values, by curve fitting via a nonlinear regression model (three-parameter logistic fit). Results are represented as the AUC ± standard error of the mean (SEM) derived from a minimum of three independent experiments (*n* ≥ 3), run in duplicate. The Grubbs test was performed to detect potential outliers, which were omitted from the dataset if applicable (*p*-value < 0.05). Dynamic photomodulation luminescence data were corrected for inter-well variability (*vide supra)* and plotted using Microsoft Excel 2024. Each experiment was performed three times (independently), and in duplicate.

**Investigation of the Nonspecific Effect at the µ-Opioid Receptor (MOR)**

**Figure S1:** Bar charts demonstrating nonspecific effects of high concentrations (10 µM and 500 nM) of the test compounds. Compounds were assayed at the MOR in the absence (panel **A**) and the presence (panel **B**) of 10 µM hydromorphone (HM). Orange bars in panel A represent responses obtained in the presence of 10 µM test compound, and dark blue bars in the presence of 500 nM test compound. Light blue bars in panel B represent responses obtained in the presence of 10 µM test compound, and green bars in the presence of 500 nM test compound. Purple bar represents the signal obtained for 10 µM of HM alone.

| *Figure S2.* |  |  |
| --- | --- | --- |
| ***n* = 1** | ***n* = 2** | ***n* = 3** |
|  |  |  |
|  |  |  |
|  |  |  |
|  |  |  |
|  |  |  |
|  |  |  |
|  |  |  |
|  |  |  |
|  |  |  |
|  |  |  |
|  |  |  |
|  |  |  |

**Figure S2:** Time–response curves for the target and reference compounds at the MOR, in absence of hydromorphone. Orange curves represent responses obtained with 10 µM test compound, dark blue curves with 100 nM test compound, and grey curves represent the blank controls. RLU: relative light units.

| *Figure S3.* |  |  |
| --- | --- | --- |
| ***n* = 1** | ***n* = 2** | ***n* = 3** |
|  |  |  |
|  |  |  |
|  |  |  |
|  |  |  |
|  |  |  |
|  |  |  |
|  |  |  |
|  |  |  |
|  |  |  |
|  |  |  |
|  |  |  |
|  |  |  |

**Figure S3:** Time–response curves for the target and reference compounds at the MOR, in presence of hydromorphone. Light blue curves represent responses obtained with 10 µM test compound, green curves with 500 nM test compound, grey curves represent the blank controls, and purple curves represent the signal of 10 µM of hydromorphone alone. RLU: relative light units.

**Impact of Storage Conditions of the Reference 5**

*Trans*-enriched photorexant (**4**) was achieved by overnight incubation at 37 °C. To exclude the potential impact of different storage conditions of the stock solutions (*cis*-state of photorexant **4** and reference **5** *vs. trans*-state of **4** overnight at 37 °C), stock solutions of reference compound **5** were kept overnight at 37 °C and results were compared to the usual pre-assay storage condition (4 °C). In three independent experiments, concentration–inhibition curves of both stock solutions tested at OX1R-βarr2 were seamlessly overlapping, indicating a negligible impact on the obtained data.

**Figure S4:** Concentration–inhibition curves of photorexant **4** (in either *cis*- or *trans*-state) and the reference compound **5** at OX1R-βarr2 (stored overnight at either 4 °C or 37 °C).

**Compatibility of the Assay with Irradiation**

Firstly, compatibility of the luciferase substrate with irradiation at 530 and 365 nm was assessed. As the mechanism of action of the nanoluciferase enzyme involves emission of light (maximal emission at 460 nm), irradiation with light of different wavelengths may impact the performance of the assay. Furthermore, the irradiation itself may affect the cells. To assess both aspects, cells transfected with the NanoBiT® system (in this case OX2R-βarr2 cells) were treated with furimazine substrate, as described before, and then irradiated for 2 min with light of either 530 or 365 nm, before continuing the luminescence measurement.

As shown in **Figure S5**, irradiation did not have a major impact on the signal, although a small decrease could be observed, potentially caused by both the irradiation and the natural degradation of the substrate during the period in which the plate was removed from the reader. This finding needs to be considered when interpreting photomodulation data.

**Figure S5:** Time–luminescence profiles of the furimazine substrate in OX2R-βarr2 cells. Green bar represents irradiation with light of 530 nm; blue bar represents irradiation with 365 nm. In each graph, 16 replicates are shown. RLU: relative light units.

Next, as irradiation of photorexant (**4**) or reference compound **5** will be done during the assay, orexin-A will already have been administered to the cells and will be present in the assay medium. To exclude a potential (negative) impact of irradiation on the orexin-A peptide and the corresponding blanks, cells expressing OX1R-βarr2 were treated with a stock solution of 1 µM of orexin-A (in-well) or a blank, which was either non-irradiated, or irradiated at either 365 or 530 nm. Based on the overlapping luminescence profiles in **Figure S6**, neither orexin-A nor the corresponding blank was substantially affected by either 365 nm or 530 nm irradiation. This implies that during dynamic photomodulation of photorexant (**4**), orexin-A itself is not expected to be affected.

**Figure S6:** Time–luminescence profiles at OX1R-βarr2 of 1 µM orexin-A (left), non-irradiated or pre-irradiated with 365 nm or 530 nm light, and the corresponding blanks (right). RLU: relative light units.

## **Dynamic Photomodulation of Photorexant**

For the dynamic photomodulation experiments, luminescence was recorded using a CLARIOstar luminometer (BMG LABTECH GmbH, Ortenberg, Germany), as it allows to pause the system mid-run. To investigate compatibility of the substrate with the irradiation, furimazine was added to cells transfected with OX2R-βarr2 and luminescence was monitored for 5 cycles, allowing the signal to rise. The plate was then removed from the luminometer and irradiated with light of either 530 nm or 365 nm for 2 min, after which luminescence measurement was continued for 5 additional cycles. To investigate compatibility of orexin-A with the irradiation, stock solutions of 1 µM orexin-A or corresponding blanks, both either non-irradiated, or irradiated at 530 nm or 365 nm, were added to OX1R-βarr2 cells after initial equilibration and luminescence was monitored for 2 h.

To assess the dynamic photomodulation of photorexant (**4**) in the OX1R-βarr2 assay, cells were, after initial equilibration, pre-incubated for approximately 5 min with two concentrations (1 µM and 100 nM, in-well concentration) of photorexant (**4**), in either its *cis*- (achieved by 10 min irradiation at 365 nm) or *trans*-state (by incubating in the dark overnight at 37° C). Similarly, the non-photoswitchable reference antagonist **5** was also assayed at 1 µM and 100 nM. Then, as deteailed before, 10 µL of orexin-A (50 nM, *in-well*) was added and the plate was placed back into the system to monitor luminescence. After 30 min, the plate was removed from the luminometer and irradiated for 3 min with either 530 nm or 365 nm light, followed by the administration of 10 or 50 nM (in-well concentration) of orexin-A, and read again in the CLARIOstar. This was repeated twice, alternating the irradiation wavelengths, resulting in either a dark(*trans*)*/cis/trans/cis* or a *cis/trans/cis/trans* photoswitching sequence. The total time outside the system was limited to 4 min to avoid substrate degradation, which may complicate data interpretation. Additionally, each individual experiment was performed on separate 96-well plates to limit the time required to administer the stock solutions after irradiation. This was essential to obtain reproducible results.

**Figure S7:** *In vitro* dynamic photomodulation of photorexant (**4**) measured in the OX1R-βarr2 assay starting from the *trans*-state with 50 nM orexin-A added after each irradiation (panel **A**), and non-photoswitchable control 5 (panel **B**). Three independent experiments (*n* = 1–3) run in duplicate are depicted. Top panels show the effects at 1 µM (purple lines) or 100 nM (blue lines) concentrations of the tested compounds. Bottom panels show a detailed view of the top panels at the 10 µM concentration. Compounds were irradiated each 30 min, starting from *trans*. The switching sequence of *trans*/*cis*/*trans*/*cis* is shown. Initial rise in signal before the 30-minute mark can be attributed to the overall moderate antagonist properties of photorexant (**4**) compared to the reference, even in the *trans*-photoform. Blue bars represent switching to *cis* with 365 nm, green bars represent switching to *trans* with 530 nm. After each irradiation, 50 nM in-well concentration orexin A was added. RLU: relative light units.

**Figure S8:** *In vitro* dynamic photomodulation of photorexant (**4**) measured in the OX1R-βarr2 assay starting from the *cis*-state with 10 nM orexin-A added after each irradiation (panel **A**), and non-photoswitchable control 5 (panel **B**). Three independent experiments (*n* = 1–3) run in duplicate are depicted. Top panels show the effects at 1 µM (purple lines) or 100 nM (blue lines) concentrations of the tested compounds. Bottom panels show a detailed view of the top panels at the 10 µM concentration. Compounds were irradiated each 30 min, starting from *cis*. The switching sequence of *cis*/*trans*/*cis*/*trans* is shown. Initial rise in signal before the 30-minute mark can be attributed to the overall moderate antagonist properties of photorexant (**4**) compared to the reference. Blue bars represent switching to *cis* with 365 nm, green bars represent switching to *trans* with 530 nm. After each irradiation, 10 nM in-well concentration orexin A was added. RLU: relative light units.

**Figure S9:** *In vitro* dynamic photomodulation of photorexant (**4**) measured in the OX1R-βarr2 assay starting from the *cis*-state with 50 nM orexin-A added after each irradiation (panel **A**), and non-photoswitchable control 5 (panel **B**). Three independent experiments (*n* = 1–3) run in duplicate are depicted. Top panels show the effects at 1 µM (purple lines) or 100 nM (blue lines) concentrations of the tested compounds. Bottom panels show a detailed view of the top panels at the 10 µM concentration. Compounds were irradiated each 30 min, starting from *cis*. The switching sequence of *cis*/*trans*/*cis*/*trans* is shown. Blue bars represent switching to *cis* with 365 nm, green bars represent switching to *trans* with 530 nm. After each irradiation, 50 nM in-well concentration orexin A was added. RLU: relative light units.

## **Neurotoxicity Characterization of Photorexant**

Neurotoxicity measurements were conducted as previously established^[6]^.

**Cell Culture General Procedures**

HT22 cells were grown in Dulbecco’s Modified Eagle Medium (DMEM, Sigma-Aldrich, Munich, Germany) supplemented with 10% (v/v) fetal calf serum (FCS) and 1% (v/v) penicillin-streptomycin. Cells were subcultured every two days and incubated at 37°C with 5% CO_2_ in a humidified incubator.

Compounds were dissolved in dimethyl sulfoxide (DMSO, Sigma-Aldrich, Munich, Germany) as stock solutions and diluted further in culture medium.

For determination of cell viability, a colorimetric 3-(4,5-dimethylthiazol-2-yl)-2,5-diphenyl tetrazolium bromide (MTT, Sigma-Aldrich, Munich, Germany) assay was used. MTT solution (5 mg/mL in PBS) was diluted 1:10 with medium and added to the wells after removal of the old medium. Cells were incubated for 3 h and then lysis buffer (10% SDS) was applied. The next day, absorbance at 560 nm was determined with a multiwell plate photometer (Tecan, SpectraMax 250).

**Neurotoxicity**

5,000 cells per well were seeded into sterile 96-well plates and incubated overnight. For the neurotoxicity assay, medium was removed and 1.25, 2.5, 5 or 10 µM of the compound diluted with medium from a 10 mM stock solution was added to the wells. 0.1% DMSO in DMEM served as a control. Cells were incubated for 24 h, when neurotoxicity was determined using a colorimetric MTT assay.

**Figure S10:** Neurotoxicity of photorexant in HT22 hippocampal neurons. Data are presented as means ± SEM from three independent experiments. Statistical analysis was performed using one-way ANOVA followed by Dunnett’s multiple comparisons test. Evaluation was carried out with GraphPad Prism 10, referring to untreated control cells. Levels of significance are indicated as **p* < 0.05; ***p* < 0.01; ****p* < 0.001; *****p* < 0.0001.

## ***In Vivo* Characterization of Photorexant**

Experiments were conducted using six young adult male C57BL/6J mice. Mice were anesthetized with isoflurane (2% induction; 1–1.5% in oxygen, maintenance doses) and placed in a David Kopf stereotaxic apparatus (Tujunga, CA, USA) in which surgical procedures and recordings were performed. Body temperature was set at 37 °C through a water-heated pad (Gaymar T/Pump, NY, USA). The skin over the midline of the scalp was sectioned and retracted. A small craniotomy was drilled over the locus coeruleus (LC) nucleus according to the atlas of Paxinos and Franklin (coordinates from Bregma: A: -5.4 mm, L: 0.9 mm lateral, H: 3.5 mm)^[7]^. Local anesthetic (lidocaine 1%) was applied to all skin incisions and pressure points.

Tungsten microelectrodes (2 MΩ World Precision Instruments, USA) were used to record the local field potential and the evoked potential in the LC (coordinates from Bregma: A, −5.4; L, 1 and depth, 4.0–4.5 mm). Unit recordings were filtered (0.3–3 kHz) and amplified via an AC preamplifier (DAM80; World Precision Instruments). The field potential was filtered (1–100 Hz) and amplified by the same preamplifier. The Pef/LH was stimulated using 120 µm diameter stainless steel bipolar electrodes (World Precision Instruments, coordinates from Bregma: A, −1.8; L, 1 and depth, 4.5–5 mm). Electrical stimulation was carried out with single square pulses (0.3 ms duration and 20–50 µA intensity, delivered at 0.5 Hz; GRASS S88 stimulator).

The signals were sampled at 10 kHz through an analog-to-digital converter (Power 1401 data acquisition unit, Cambridge Electronic Design, Cambridge, UK) and fed into a PC for off-line analysis with Spike 2 software (Cambridge Electronic Design).

The experimental protocol consisted of a 30 s period of spontaneous activity, followed by 30 electric pulses delivered at the PeF area (0.5Hz for 60 s). After the basal recording, *trans*-irradiated or non-irradiated photorexant (**4**) was injected locally into the LC (1 µl, 200 nM). The recording and stimulation were maintained for 30 minutes after the drug was administered.

Spike2 software was used to perform statistical calculations including peristimulus time histograms (PSTHs), which were calculated using 1-ms binwidth, and the evoked potential in the field potential. Statistical analyses were calculated with one-way ANOVA for unpaired data. Differences were considered statistically significant at a level of 95% (*p* < 0.05). All data are indicated as mean ± SEM.

All experimental procedures complied with European legislation (Directive 2010/63/EU) and were approved by the Ethics Committee of the Autonomous University of Madrid and the Regional Government of Madrid (PROEX: 181.6/21).

# **Photochemistry**

## **General Methods**

UV/Vis experiments were performed with a Varian Cary 50 Bio UV/Vis spectrophotometer using Hellma (Type 100-QS) quartz glass Suprasil cuvettes (10 mm light path). Samples were prepared at a concentration of 25-37.5 µM in DMSO:PBS (pH 7.4, 1:1). Absorption spectra were measured at room temperature and analyzed using CaryWinUV software. Samples were irradiated from a distance of 5 cm using LEDs of Seoulviosys, Cree, Lumileds and LedEngin emitting the wavelengths 365 nm, 385 nm, 400 nm, 420 nm, 433 nm, 451 nm, 505 nm and 530 nm (FWHM ≈ 20-25 nm). Only spectra where irradiation of the solutions with the respective wavelength for 60 seconds yielded maximal conversion to the corresponding equilibrium are shown. Wavelengths not allowing for full conversion in the given time frame were excluded. Thermal relaxation was measured by switching the photochromic compounds into the thermodynamically meta-stable *cis*-isomer and observing the decrease in isomer ratio over a certain time period. Thermal half-lives were calculated through non-linear regression, assuming first order exponential decay. Stability against photofatigue was determined by repeated irradiation of the sample with the respective *cis-* and *trans*-wavelength and confirming that the absorption remained constant at equilibrium. Photoisomer ratios were determined by liquid chromatography–mass spectrometry (LC-MS) measurement with the detector wavelength set to the isosbestic point (the wavelength, where the absorption coefficient of both isomers is equal).

## **Photocharacterization Data**

**Figure S11:** Photocharacterization of compound **1a** measured in a 25 µM solution of 1:1 PBS, DMSO at 23 °C. **A**. Absorption spectra recorded after irradiation with LEDs of different wavelengths. **B**. Absorption spectra recorded after irradiation with the best *cis-* and *trans-*switching wavelengths. **C**. Thermal half-life was determined by measuring the increase in absorbance after switching to the *cis*-isomer. **D**. Stability against photofatigue was confirmed by repeated irradiation with the *cis-* and *trans-*photoisomerization wavelengths, respectively.

**Figure S12:** Photocharacterization of compound **1b** measured in a 25 µM solution of 1:1 PBS, DMSO at 23 °C. **A**. Absorption spectra recorded after irradiation with LEDs of different wavelengths. **B**. Absorption spectra recorded after irradiation with the best *cis-* and *trans-*switching wavelengths. **C**. Thermal half-life was determined by measuring the increase in absorbance after switching to the *cis*-isomer. **D**. Stability against photofatigue was confirmed by repeated irradiation with the *cis-* and *trans-*photoisomerization wavelengths, respectively.

**Figure S13:** Photocharacterization of compound **1c** measured in a 25 µM solution of 1:1 PBS, DMSO at 23 °C. **A**. Absorption spectra recorded after irradiation with LEDs of different wavelengths. **B**. Absorption spectra recorded after irradiation with the best *cis-* and *trans-*switching wavelengths. **C**. Thermal half-life was determined by measuring the increase in absorbance after switching to the *cis*-isomer. **D**. Stability against photofatigue was confirmed by repeated irradiation with the *cis-* and *trans-*photoisomerization wavelengths, respectively.

**Figure S14:** Photocharacterization of compound **2a** measured in a 25 µM solution of 1:1 PBS, DMSO at 23 °C. **A**. Absorption spectra recorded after irradiation with LEDs of different wavelengths. **B**. Absorption spectra recorded after irradiation with the best *cis-* and *trans-*switching wavelengths. **C**. Thermal half-life was determined by measuring the increase in absorbance after switching to the *cis*-isomer. **D**. Stability against photofatigue was confirmed by repeated irradiation with the *cis-* and *trans-*photoisomerization wavelengths, respectively.

**Figure S15:** Photocharacterization of compound **2b** measured in a 25 µM solution of 1:1 PBS, DMSO at 23 °C. **A**. Absorption spectra recorded after irradiation with LEDs of different wavelengths. **B**. Absorption spectra recorded after irradiation with the best *cis-* and *trans-*switching wavelengths. **C**. Thermal half-life was determined by measuring the increase in absorbance after switching to the *cis*-isomer. **D**. Stability against photofatigue was confirmed by repeated irradiation with the *cis-* and *trans-*photoisomerization wavelengths, respectively.

**Figure S16:** Photocharacterization of compound **2c** measured in a 25 µM solution of 1:1 PBS, DMSO at 23 °C. **A**. Absorption spectra recorded after irradiation with LEDs of different wavelengths. **B**. Absorption spectra recorded after irradiation with the best *cis-* and *trans-*switching wavelengths. **C**. Thermal half-life was determined by measuring the increase in absorbance after switching to the *cis*-isomer. **D**. Stability against photofatigue was confirmed by repeated irradiation with the *cis-* and *trans-*photoisomerization wavelengths, respectively.

**Figure S17:** Photocharacterization of compound **3a** measured in a 25 µM solution of 1:1 PBS, DMSO at 23 °C. **A**. Absorption spectra recorded after irradiation with LEDs of different wavelengths. **B**. Absorption spectra recorded after irradiation with the best *cis-* and *trans-*switching wavelengths. **C**. Thermal half-life was determined by measuring the increase in absorbance after switching to the *cis*-isomer. **D**. Stability against photofatigue was confirmed by repeated irradiation with the *cis-* and *trans-*photoisomerization wavelengths, respectively.

**Figure S18:** Photocharacterization of compound **3b** measured in a 25 µM solution of 1:1 PBS, DMSO at 23 °C. **A**. Absorption spectra recorded after irradiation with LEDs of different wavelengths. **B**. Absorption spectra recorded after irradiation with the best *cis-* and *trans-*switching wavelengths. **C**. Thermal half-life was determined by measuring the increase in absorbance after switching to the *cis*-isomer. **D**. Stability against photofatigue was confirmed by repeated irradiation with the *cis-* and *trans-*photoisomerization wavelengths, respectively.

**Figure S19:** Photocharacterization of compound **3c** measured in a 25 µM solution of 1:1 PBS, DMSO at 23 °C. **A**. Absorption spectra recorded after irradiation with LEDs of different wavelengths. **B**. Absorption spectra recorded after irradiation with the best *cis-* and *trans-*switching wavelengths. **C**. Thermal half-life was determined by measuring the increase in absorbance after switching to the *cis*-isomer. **D**. Stability against photofatigue was confirmed by repeated irradiation with the *cis-* and *trans-*photoisomerization wavelengths, respectively.

**Figure S20:** Photocharacterization of compound **4** measured in a 37.5 µM solution of 1:1 PBS, DMSO at 23 °C. **A**. Absorption spectra recorded after irradiation with LEDs of different wavelengths. **B**. Absorption spectra recorded after irradiation with the best *cis-* and *trans-*switching wavelengths. **C**. Thermal half-life was determined by measuring the increase in absorbance after switching to the *cis*-isomer. **D**. Stability against photofatigue was confirmed by repeated irradiation with the *cis-* and *trans-*photoisomerization wavelengths, respectively.

## **Determination of PSS Ratios**

**Figure S21:** PSS Ratio of compound **1a** under 365 nm irradiation

**Figure S22:** PSS Ratio of compound **1a** under 420 nm irradiation

**Figure S23:** PSS Ratio of compound **1b** under 365 nm irradiation

**Figure S24:** PSS Ratio of compound **1b** under 420 nm irradiation

**Figure S25:** PSS Ratio of compound **1c** under 365 nm irradiation

**Figure S26:** PSS Ratio of compound **1c** under 365 nm irradiation

**Figure S27:** PSS Ratio of compound **2a** under 365 nm irradiation

**Figure S28:** PSS Ratio of compound **2a** under 420 nm irradiation

**Figure S29:** PSS Ratio of compound **2b** under 365 nm irradiation

**Figure S30:** PSS Ratio of compound **2b** under 420 nm irradiation

**Figure S31:** PSS Ratio of compound **2c** under 365 nm irradiation

**Figure S32:** PSS Ratio of compound **2c** under 420 nm irradiation

**Figure S33:** PSS Ratio of compound **3a** under 365 nm irradiation

**Figure S34:** PSS Ratio of compound **3a** under 420 nm irradiation

**Figure S35:** PSS Ratio of compound **3b** under 365 nm irradiation

**Figure S36:** PSS Ratio of compound **3b** under 420 nm irradiation

**Figure S37:** PSS Ratio of compound **3c** under 365 nm irradiation

**Figure S38:** PSS Ratio of compound **3c** under 420 nm irradiation

**Figure S39:** PSS Ratio of compound **4** under 365 nm irradiation

**Figure S40:** PSS Ratio of compound **4** under 530 nm irradiation

# **Synthetic Procedures**

## **General Methods**

All reagents and solvents were purchased from the following commercial suppliers: ABCR, BLD-Pharm, Enamine, Sigma-Aldrich, Thermo Fisher and were used without further purification.

Reaction progress was monitored using analytical thin-layer chromatography (TLC) on precoated silica gel 60 GF254 plates (Macherey Nagel GmbH & Co. KG, Düren, Germany). Detection was carried out by irradiation and consequent fluorescence quenching at 254 nm or excitation at 365 nm. Compounds were purified by column chromatography using silica gel 60 (60 Å pore size, 40–63 μm; Macherey Nagel GmbH & Co. KG, Düren, Germany) as the stationary phase. Reverse phase column chromatography was performed on an Interchim Puri Flash 430 (Ultra Performance Flash Purification) instrument connected to an Interchim Flash ELSD. FlashPure 4 g C18 columns were used.

Nuclear magnetic resonance (NMR) spectra were measured on a Bruker AV-400 NMR instrument (Bruker, Karlsruhe, Germany) in deuterated solvents (DMSO-d_6_, CDCl_3_, CD_2_Cl_2_). Chemical shifts are expressed in ppm relative to DMSO-d_6_ (^1^H: δ=2.50 ppm; ^13^C: δ=39.52 ppm) CDCl_3_ (^1^H: δ=7.26 ppm; ^13^C: δ=77.16 ppm) and CD_2_Cl_2_ (^1^H: δ=5.32 ppm; ^13^C: δ=53.84 ppm).

LC-MS measurements for verification and purity of the compounds were performed with a Shimadzu LC/MS system, comprising a DGU-20A3R controller, pump LC-20AB, degasser DGU-20A, and SPD-20A UV/VIS detector. Compounds were dissolved in MeOH and filtered through syringe filters prior to measurement. As stationary phase, a Phenomenex Synergi 4U fusion-RP 80 Å (150×4.6 mm) column was used. As a mobile phase, a gradient of MeOH/H_2_O (both containing 0.1 % formic acid (FA)) was used. Method: flow rate: 1.0 mL/min; detection: 254 nm; scan range: 100–1000 m/z; solvent A: H_2_O (0.1 % FA); solvent B: MeOH (0.1 % FA); 0–8 min 5 %→90 % B, 8–13 min 90 % B, 13–14 min 90 %→5 % B, 14–18 min 5 % B. The purity of all final target compounds was confirmed to be ≥95 %. Purities were not tested at a particular PSS and therefore show an arbitrary ratio of *cis-* and *trans*-isomers present under ambient light conditions, as indicated by green highlight. If a compound was detected at multiple times due to *cis/trans* isomerism or different protonation species, then all detected retention times are reported. ESI ionization was accomplished by a downstream Shimadzu LCMS-2020 mass spectrometer.

High-resolution mass spectra were recorded on a Bruker micrOTOF-Q III with positive ESI for ion generation.

## **Synthetic Procedures**

***Tert*-butyl 1,4-diazepane-1-carboxylate (7)**

Prepared according to literature procedure^[8]^ using 1.0 g (10.0 mmol) homopiperazine and 1.13 g (5.2 mmol, 0.52 eq) di-tert-butyl decarbonate (Boc_2_O). **Yield:** 1.0 g, quant.

Spectral data in accordance with literature report.

***Tert*-butyl 4-(5-chlorobenzo[*d*]oxazol-2-yl)-1,4-diazepane-1-carboxylate (8)**

*Tert*-butyl 1,4-diazepane-1-carboxylate (1.0 g, 5.0 mmol) and 2,5-dichlorobenzoxazole (950 mg, 5.0 mmol, 1 eq) were dissolved in 15 ml DMF. 3 ml Et_3_N were added and the reaction mixture was stirred at 90 °C for 20 hours. The mixture was diluted with 50 ml H_2_O and extracted with 4x10 ml DCM. The organic phase was washed with water and brine and the solvent removed in vacuo. Flash column chromatography (2/1 cyclohexane, ethyl acetate) afforded the product as an off-white solid. **Yield**: 1.16 g, 66 %

^1^H NMR (400 MHz, CDCl_3_) δ 7.29 (d, *J* = 2.1 Hz, 1H), 7.13 (d, *J* = 8.4 Hz, 1H), 6.95 (dd, *J* = 8.4, 2.1 Hz, 1H), 3.82 – 3.75 (m, 2H), 3.73 – 3.68 (m, 2H), 3.65 – 3.58 (m, 2H), 3.49 – 3.34 (m, 2H), 2.01 (p, *J* = 6.1 Hz, 2H), 1.41 (d, *J* = 2.4 Hz, 9H).

^13^C NMR (101 MHz, CDCl_3_) δ 162.8, 147.6, 144.8, 129.4, 120.3, 116.3, 109.3, 80.1, 49.5, 47.7, 46.6, 46.2, 28.5, 26.6.

ESI-MS [C17H22ClN3O3+H]^+^: m/z calcd. 352.13, found 352.20, t = 10.63 min

**5-chloro-2-(1,4-diazepan-1-yl)benzo[*d*]oxazole (9)**

*Tert*-butyl 4-(5-chlorobenzo[d]oxazol-2-yl)-1,4-diazepane-1-carboxylate (508 mg, 1.4 mmol) was dissolved in 10 ml DCM and 1 ml TFA was added. The reaction was stirred at room temperature for 5 h. The solution was reduced *in vacuo* and 1M NaOH was added. The formed precipitate was extracted with DCM, the organic phase was extracted again with 1 N HCl, adjusted to pH 12 with 1 N NaOH and extracted with DCM. The product was obtained as a brown oil. **Yield:** 323 mg, 89 %

^1^H NMR (400 MHz, CDCl_3_) δ 7.29 (d, *J* = 2.1 Hz, 1H), 7.12 (d, *J* = 8.4 Hz, 1H), 6.94 (dd, *J* = 8.4, 2.1 Hz, 1H), 3.87 – 3.70 (m, 4H), 3.12 – 3.01 (m, 2H), 2.97 – 2.84 (m, 2H), 2.02 – 1.87 (m, 2H).

^13^C NMR (101 MHz, CDCl_3_) δ 163.3, 147.6, 145.1, 129.3, 120.0, 116.2, 109.2, 51.3, 49.3, 48.8, 47.5, 30.5.

ESI-MS [C12H14ClN3O+H]^+^: m/z calcd. 252.08, found 352.25, t = 6.39 min

**2-(phenyldiazenyl)benzoic acid (10a)**

Nitrosobenzene (300 mg, 2.8 mmol) and 2-Aminobenzoic acid (425 mg, 3.1 mmol, 1.1 eq) were dissolved in 10 ml HOAc and 10 ml EtOH. The reaction mixture was stirred at room temperature for 2 days and reduced *in vacuo*. The crude product was dissolved in EtOAc and extracted with 1 N NaOH. The aqueous phase was acidified with 1 N HCl, extracted with EtOAc and the solvent removed *in vacuo*. The crude product was purified via flash column chromatography (DCM, 5 % MeOH, 2 % HOAc). The product was obtained as a brown solid. **Yield:** 140 mg, 22 %

^1^H NMR (400 MHz, DMSO) δ 13.13 (s, 1H), 7.90 – 7.83 (m, 2H), 7.83 – 7.76 (m, 1H), 7.70 – 7.65 (m, 1H), 7.64 – 7.53 (m, 5H).

^13^C NMR (101 MHz, DMSO) δ 168.2, 152.0, 150.8, 131.8, 131.7, 130.4, 130.3, 129.5, 129.2, 122.7, 118.0.

ESI-MS [C13H10N2O2+H]^+^: m/z calcd. 227.07, found 227.10, t = 9.88 min

**3-(phenyldiazenyl)benzoic acid (10b)**

Nitrosobenzene (300 mg, 2.8 mmol) and 3-Aminobenzoic acid (420 mg, 3.1 mmol, 1.1 eq) were dissolved in 10 ml HOAc and the reaction mixture was stirred at room temperature overnight. The mixture was diluted with 50 ml H_2_O and extracted with EtOAc. The organic phase was washed with H_2_O and sat. NaCl and the solvent removed in vacuo. The product was obtained as an orange solid and used without further purification. **Yield**: 645 mg, 84 %

^1^H NMR (400 MHz, DMSO) δ 13.29 (s, 1H), 8.41 – 8.35 (m, 1H), 8.19 – 8.10 (m, 2H), 7.98 – 7.90 (m, 2H), 7.80 – 7.72 (m, 1H), 7.67 – 7.57 (m, 3H).

^13^C NMR (101 MHz, DMSO) δ 166.7, 151.9, 151.8, 132.2, 131.9, 131.8, 130.0, 129.5, 127.3, 122.7, 122.2.

ESI-MS [C13H10N2O2+H]^+^: m/z calcd. 227.07, found 226.95, t = 10.55 min

**4-(phenyldiazenyl)benzoic acid (10c)**

Prepared analogously to 3-(phenyldiazenyl)benzoic acid from Nitrosobenzene (300 mg, 2.8 mmol) and 4-Aminobenzoic acid (410 mg, 3.0 mmol, 1.0 eq). The product was purified by recrystallization from EtOAc. **Yield**: 401 mg, 63 %.

^1^H NMR (400 MHz, DMSO) δ 13.22 (s, 1H), 8.20 – 8.10 (m, 2H), 8.01 – 7.89 (m, 4H), 7.68 – 7.56 (m, 3H).

^13^C NMR (101 MHz, DMSO) δ 166.7, 154.3, 151.9, 132.8, 132.2, 130.6, 129.6, 122.8, 122.5.

ESI-MS [C13H10N2O2+H]^+^: m/z calcd. 227.07, found 226.90, t = 10.64 min

**2-nitrosobenzoic acid**

2-Aminobenzoic acid (1.53 g, 11.2 mmol) was dissolved in 50 ml DCM and 10 ml methanol. To this was added Oxone (14.3 g, 2.1 eq) dissolved in 100 ml H_2_O. The mixture was stirred at room temperature for 3.5 hours and acidified with 1 N HCl. The resulting precipitate was filtered and used without further purification or characterization in the following reaction.

ESI-MS [C7H5NO3+H]^+^: m/z calcd. 152.03, found 151.90, t = 7.18 min

**2-((4-methoxyphenyl)diazenyl)benzoic acid (10d)**

2-nitrosobenzoic acid (176 mg, 1.2 mmol) and 4-methoxyaniline (142 mg, 1.2 mmol, 1.0 eq.) were dissolved in 2 ml HOAc. The mixture was stirred at room temperature for 24 hours, acidified with 1 N HCl and extracted with EtOAc. The product was purified by flash column chromatography (1/1 EtOAc, cyclohexane). **Yield:** 72 mg, 24 %

^1^H NMR (400 MHz, CDCl_3_) δ 13.44 (s, 1H), 8.43 (dd, *J* = 7.6, 1.8 Hz, 1H), 8.02 (dd, *J* = 7.9, 1.5 Hz, 1H), 7.93 – 7.85 (m, 2H), 7.73 – 7.58 (m, 2H), 7.13 – 7.03 (m, 2H), 3.94 (s, 3H).

^13^C NMR (101 MHz, CDCl_3_) δ 166.5, 164.3, 149.6, 145.6, 133.7, 132.9, 131.8, 126.4, 126.2, 115.8, 115.1, 55.9.

ESI-MS [C14H12N2O3+H]^+^: m/z calcd. 257.08, found 257.15, t = 9.81 min

**General procedure I for synthesis of amine containing photoswitches**

The respective aminomethylaniline or Boc-protected aminoethylaniline were dissolved in acetic acid and nitrosobenzene (1.2-1.5 eq) was added. The mixture was stirred at room temperature overnight, basified with 1 N NaOH and extracted with EtOAc. The product was purified by flash column chromatography (EtOAc, 10% MeOH, 1% Et_3_N for free amines; Cyclohexane, 20 % EtOAc for Boc-protected amines)

***tert*-butyl (2-(phenyldiazenyl)phenethyl)carbamate (11a)**

Prepared according to general procedure I from *tert*-butyl (2-aminophenethyl)carbamate (48 mg, 0.20 mmol). **Yield**: 36 mg, 54 %

^1^H NMR (400 MHz, CDCl_3_, cis and trans isomer signals observed) δ 7.93 – 7.78 (m, 2H), 7.67 – 7.58 (m, 1H), 7.50 – 7.39 (m, 3H), 7.37 – 7.33 (m, 1H), 7.32 – 7.23 (m, 2H), 4.71 (s, 1H), 3.47 – 3.35 (m, 2H), 3.33 – 3.18 (m, 2H), 1.32 (s, 9H).

^13^C NMR (101 MHz, CDCl_3_, cis and trans isomer signals observed) δ 156.0, 153.0, 150.7, 139.2, 131.4, 131.2, 131.2, 130.6, 129.3, 128.9, 127.9, 127.5, 123.2, 120.7, 115.8, 79.1, 42.4, 31.9, 28.5, 28.5.

ESI-MS [C19H23N3O2+H]^+^: m/z calcd. 326.18, found 326.10, [C19H23N3O2+Na]^+^: m/z calcd. 348.17, found 348.05, t = 10.02 min, 10.99 min

***tert*-butyl (3-(phenyldiazenyl)phenethyl)carbamate (11b)**

Prepared according to general procedure I from *tert*-butyl (3-aminophenethyl)carbamate (50 mg, 0.20 mmol). **Yield**: 50 mg, 76 %

^1^H NMR (400 MHz, CDCl_3_) δ 7.93 – 7.84 (m, 2H), 7.78 – 7.68 (m, 2H), 7.52 – 7.37 (m, 4H), 7.30 – 7.24 (m, 1H), 4.57 (s, 1H), 3.49 – 3.30 (m, 2H), 2.87 (t, *J* = 7.1 Hz, 2H), 1.40 (s, 9H).

^13^C NMR (101 MHz, CDCl_3_) δ 156.0, 153.0, 152.8, 140.3, 131.6, 131.1, 129.4, 129.2, 123.0, 122.9, 121.5, 79.4, 41.8, 36.2, 28.5.

ESI-MS [C19H23N3O2+H]^+^: m/z calcd. 326.18, found 326.05, [C19H23N3O2+Na]^+^: m/z calcd. 348.17, found 348.10, t = 9.87 min, 10.88 min

***tert*-butyl (4-(phenyldiazenyl)phenethyl)carbamate (11c)**

Prepared according to general procedure I from *tert*-butyl (4-aminophenethyl)carbamate (54 mg, 0.23 mmol). **Yield**: 60 mg, 81 %

^1^H NMR (400 MHz, CDCl_3_, *cis* and *trans* isomer signals observed) δ 7.94 – 7.83 (m, 4H), 7.56 – 7.43 (m, 3H), 7.38 – 7.31 (m, 2H), 4.56 (s, 1H), 3.50 – 3.37 (m, 2H), 2.89 (t, *J* = 7.0 Hz, 2H), 1.44 (s, 9H).

^13^C NMR (101 MHz, CDCl_3_, *cis* and *trans* isomer signals observed) δ 156.0, 152.8, 151.6, 142.6, 131.0, 129.7, 129.2, 123.2, 122.9, 121.2, 120.5, 79.5, 41.8, 36.2, 28.5.

ESI-MS [C19H23N3O2+H]^+^: m/z calcd. 326.18, found 326.05, [C19H23N3O2+Na]^+^: m/z calcd. 348.17, found 348.00, t = 9.85 min, 10.90 min

**(2-(phenyldiazenyl)phenyl)methanamine (12a)**

Prepared according to general procedure I from 2-(aminomethyl)aniline (598 mg, 4.89 mmol). **Yield**: 291 mg, 28 %

^1^H NMR (400 MHz, CDCl_3_) δ 7.97 – 7.86 (m, 2H), 7.76 – 7.68 (m, 1H), 7.57 – 7.41 (m, 5H), 7.39 – 7.32 (m, 1H), 4.31 (s, 2H), 1.97 (s, 2H).

^13^C NMR (101 MHz, CDCl_3_) δ 152.9, 150.1, 142.5, 131.6, 131.3, 129.3, 127.9, 123.1, 120.7, 116.0, 43.6.

ESI-MS [C13H13N2+H]^+^: m/z calcd. 212.11, found 212.30, t = 6.87 min

**(3-(phenyldiazenyl)phenyl)methanamine (12b)**

Prepared according to general procedure I from 3-(aminomethyl)aniline (623 mg, 5.10 mmol). **Yield**: 325 mg, 60 % (87 % LC-MS purity)

^1^H NMR (400 MHz, CDCl_3_) δ 7.96 – 7.90 (m, 2H), 7.90 – 7.86 (m, 1H), 7.84 – 7.79 (m, 1H), 7.55 – 7.42 (m, 5H), 3.98 (s, 2H), 1.63 (s, 2H).

^13^C NMR (101 MHz, CDCl_3_) δ 153.1, 152.8, 144.6, 131.1, 129.8, 129.4, 129.2, 123.0, 122.1, 120.9, 46.4.

ESI-MS [C13H13N2+H]^+^: m/z calcd. 212.11, found 212.20, t = 2.08 min, 6.88 min

**(4-(phenyldiazenyl)phenyl)methanamine (12c)**

Prepared according to general procedure I from 4-(aminomethyl)aniline (500 mg, 4.09 mmol). **Yield**: 240 mg, 28 %

^1^H NMR (400 MHz, CDCl_3_) δ 7.96 – 7.86 (m, 4H), 7.58 – 7.42 (m, 5H), 3.96 (s, 2H), 1.55 (s, 2H).

^13^C NMR (101 MHz, CDCl_3_) δ 152.8, 151.8, 146.6, 131.0, 129.2, 127.8, 123.2, 122.9, 46.4.

ESI-MS [C13H13N2+H]^+^: m/z calcd. 212.11, found 212.20, t = 2.08 min, 6.19-7.06 min

**General procedure II for amide couplings**

The acid (1 eq), amine (1 eq) and HBTU (1.5 eq) were dissolved in DMF. DIPEA (5 eq) was added and the mixture was stirred at room temperature overnight. The mixture was diluted with water, extracted with ethyl acetate and the organic phase was washed with sat. NaCl. After removal of the solvent *in vacuo* the product was purified by flash column chromatography.

In the case of Boc-protected amines a deprotection with 10 % TFA in DCM was performed first. The solvent and TFA were removed *in vacuo* and the amine was used without further purification in the amide coupling.

**(4-(5-chlorobenzo[d]oxazol-2-yl)-1,4-diazepan-1-yl)(2-(phenyldiazenyl)phenyl)-methanone (1a)**

Ethylchloroformate (12 µl, 0.13 mmol) was dissolved in 0.5 ml dry DCM and cooled to 0°C. To this was added 2-(phenyldiazenyl)benzoic acid (30 mg, 0.13 mmol, 1.0 eq) dissolved in 1 ml dry DCM followed by triethylamine (18.5 µl, 0.13 mmol, 1.0 eq). The reaction mixture was stirred at 0 °C for 2 hours and 5-chloro-2-(1,4-diazepan-1-yl)benzo[d]oxazole (33.3 mg, 0.13 mmol, 1.0 eq) dissolved in 0.5 ml DCM was added. The mixture was stirred at room temperature overnight and diluted with 20 ml DCM. The organic phase was washed with NaHCO_3_ and sat. NaCl and the solvent was removed *in vacuo*. Flash column chromatography (70/30 EtOAc/Cyclohexane) afforded the product as an orange solid. **Yield**: 50 mg, 82 %

^1^H NMR (400 MHz, CD_2_Cl_2_, Rotamers and *cis*–*trans* isomer signals observed) δ 7.89 – 7.70 (m, 2.6H), 7.58 – 7.49 (m, 1.3), 7.49 – 7.42 (m, 1.8H), 7.42 – 7.32 (m, 1.8H), 7.30 – 7.22 (m, 0.8H), 7.21 – 7.15 (m, 1.1H), 7.15 – 7.11 (m, 0.6H), 7.10 – 7.06 (m, 0.3H), 7.05 – 6.96 (m, 1.1H), 6.95 – 6.86 (m, 0.5H), 6.21 – 6.08 (m, 0.1H), 4.15 – 3.60 (m, 6H), 3.60 – 3.40 (m, 1.5H), 3.40 – 3.25 (m, 0.8H), 2.28 – 2.06 (m, 1.1H), 2.06 – 1.95 (m, 0.1H), 1.82 – 1.58 (m, 1.7H+H_2_O).

^13^C NMR (101 MHz, CD_2_Cl_2_, Rotamers and *cis*–*trans* isomer signals observed) δ 169.8 (C), 169.8 (C), 163.3 (C), 162.9 (C), 152.8 (C), 148.7 (C), 148.2 (C), 148.0 (C), 145.5 (C), 145.3 (C), 136.7 (C), 136.6 (C), 131.9 (CH), 131.9 (CH), 131.7 (CH), 131.7 (CH), 130.1 (CH), 130.0 (CH), 129.6 (CH), 129.5 (CH), 129.5, 129.4, 129.1 (CH), 127.6 (CH), 127.5 (CH), 123.2 (CH), 120.7 (CH), 120.6 (CH), 120.4 (CH), 120.4 (CH), 117.9 (CH), 117.6 (CH), 116.3 (CH), 116.3 (CH), 109.7 (CH), 109.6 (CH), 49.8 (CH_2_), 49.2 (CH_2_), 49.0 (CH_2_), 48.9 (CH_2_), 48.8 (CH_2_), 47.1 (CH_2_), 46.3 (CH_2_), 44.6 (CH_2_), 28.6 (CH_2_), 27.2 (CH_2_).

ESI-MS [C25H22ClN5O2+H]^+^: m/z calcd. 460.15, found 460.05, t = 10.44 min, 10.75 min

HR-ESI-MS [C25H22ClN5O2+H]^+^: m/z calcd. 460.15348, found 460.15576

**(4-(5-chlorobenzo[d]oxazol-2-yl)-1,4-diazepan-1-yl)(3-(phenyldiazenyl)phenyl)-methanone (1b)**

Prepared according to general procedure II from 3-(phenyldiazenyl)benzoic acid (50 mg, 0.22 mmol) and 5-chloro-2-(1,4-diazepan-1-yl)benzo[d]oxazole (60 mg, 0.24 mmol, 1.1 eq). Flash column chromatography (70/30 EtOAc/Cyclohexane). **Yield:** 86 mg, 85 %

^1^H NMR (400 MHz, CD_2_Cl_2_, Rotamers and *cis*–*trans* isomer signals observed) δ 8.08 – 7.82 (m, 3.2H), 7.80 – 7.75 (m, 0.5H), 7.65 – 7.40 (m, 4.2H), 7.39 – 7.08 (m, 2.8H), 7.03 – 6.88 (m, 1H), 6.88 – 6.78 (m, 0.2H), 6.63 – 6.53 (m, 0.1H), 4.04 – 3.89 (m, 1.8H), 3.89 – 3.58 (m, 5.3H), 3.57 – 3.44 (m, 1H), 3.28 – 3.17 (m, 0.1H), 3.09 (m, 0.1H), 2.23 – 2.09 (m, 1H), 2.08 – 1.98 (m, 0.2H), 1.95 – 1.77 (m, 1.2H).

^13^C NMR (101 MHz, CD_2_Cl_2_, Rotamers and *cis*–*trans* isomer signals observed) δ 170.6 (C), 162.9 (C), 162.5 (C), 153.4 (C), 152.4 (C), 152.4 (C), 147.7 (C), 144.9 (C), 137.5 (C), 131.5 (CH), 129.5 (CH), 129.4 (CH), 129.2 (CH), 129.1 (CH), 128.9 (CH), 128.7 (CH), 127.5 (CH), 124.2 (CH), 123.9 (CH), 123.0 (CH), 120.6 (CH), 120.3 (CH), 120.2 (CH), 120.0 (CH), 116.0 (CH), 109.3 (CH), 49.6 (CH_2_), 49.1 (CH_2_), 48.8 (CH_2_), 47.9 (CH_2_), 46.7 (CH_2_), 46.6 (CH_2_), 45.1 (CH_2_), 28.4 (CH_2_), 26.4 (CH_2_).

ESI-MS [C25H22ClN5O2+H]^+^: m/z calcd. 460.15, found 460.05, t = 10.18 min, 11.04 min

HR-ESI-MS [C25H22ClN5O2+H]^+^: m/z calcd. 460.15348, found 460.15527

**(4-(5-chlorobenzo[d]oxazol-2-yl)-1,4-diazepan-1-yl)(4-(phenyldiazenyl)phenyl)-methanone (1c)**

Prepared according to general procedure II from 4-(phenyldiazenyl)benzoic acid (50 mg, 0.22 mmol) and 5-chloro-2-(1,4-diazepan-1-yl)benzo[d]oxazole (59 mg, 0.24 mmol, 1.1 eq). Obtained pure compound without further purification. **Yield:** 98 mg, 95 %

^1^H NMR (400 MHz, CD_2_Cl_2_, Rotamers and *cis*–*trans* isomer signals observed) δ 8.03 – 7.82 (m, 3,8H), 7.63 – 7.42 (m, 3.8H), 7.41 – 7.11 (m, 3.2H), 7.09 – 6.92 (m, 1H), 6.88 – 6.75 (m, 0.1H), 4.01 – 3.88 (m, 1.9H), 3.88 – 3.62 (m, 5.2H), 3.57 – 3.41 (m, 1H), 2.22 – 2.06 (m, 0.1H), 1.97 – 1.77 (m, 1H).

^13^C NMR (101 MHz, CD_2_Cl_2_ Rotamers and *cis*–*trans* isomer signals observed) δ 171.0, 163.3, 162.9, 153.8, 153.3, 152.9, 148.1, 145.4, 139.0, 131.9, 129.6, 129.2, 128.0, 127.8, 127.7, 123.3, 120.8, 120.6, 120.4, 116.4, 116.3, 109.7, 50.0, 49.4, 49.2, 48.3, 47.1, 46.9, 45.4, 28.8, 26.7.

ESI-MS [C25H22ClN5O2+H]^+^: m/z calcd. 460.15, found 460.05, t = 10.20 min, 11.10 min

HR-ESI-MS [C25H22ClN5O2+H]^+^: m/z calcd. 460.15348, found 460.15402

**5-methyl-*N*-(2-(phenyldiazenyl)phenethyl)-2-(2*H*-1,2,3-triazol-2-yl)benzamide (2a)**

Prepared according to general procedure II from 5-methyl-2-(2*H*-1,2,3-triazol-2-yl)benzoic acid (24 mg, 0.12 mmol, 1.1 eq) and *tert*-butyl (2-(phenyldiazenyl)phenethyl)carbamate (34 mg, 0.10 mmol). Flash column chromatography (1/1 EtOAc, Cyclohexane). **Yield:** 40 mg, 93 %

^1^H NMR (400 MHz, CDCl_3_, *cis*–*trans* isomers observed) δ 7.77 – 7.70 (m, 2H), 7.65 – 7.60 (m, 1H), 7.60 – 7.55 (m, 2H), 7.48 – 7.42 (m, 1H), 7.41 – 7.33 (m, 3H), 7.33 – 7.21 (m, 3H), 7.20 – 7.17 (m, 1H), 7.16 – 7.12 (m, 1H), 6.15 – 6.01 (m, 1H), 3.61 (td, *J* = 6.8, 5.4 Hz, 2H), 3.25 (t, *J* = 6.8 Hz, 2H), 2.23 (s, 3H).

^13^C NMR (101 MHz, CDCl_3_, *cis*–*trans* isomers observed) δ 167.7, 152.8, 150.6, 139.0, 138.9, 135.7, 135.6, 134.8, 131.5, 131.3, 131.2, 131.2, 131.1, 130.5, 129.8, 129.6, 129.2, 128.9, 128.0, 127.5, 126.7, 124.2, 123.0, 120.7, 117.1, 115.9, 41.8, 40.4, 31.0, 29.8, 29.5, 22.8, 21.1, 21.0, 14.2.

ESI-MS [C24H22N6O+H]^+^: m/z calcd. 411.19, found 411.10, t = 9.56 min, 10.55 min

HR-ESI-MS [C24H22N6O+H]^+^: m/z calcd. 411.19279, found 411.19409

**5-methyl-*N*-(3-(phenyldiazenyl)phenethyl)-2-(2*H*-1,2,3-triazol-2-yl)benzamide (2b)**

Prepared according to general procedure II from 5-methyl-2-(2*H*-1,2,3-triazol-2-yl)benzoic acid (36 mg, 0.18 mmol, 1.1 eq) and *tert*-butyl (3-(phenyldiazenyl)phenethyl)carbamate (50 mg, 0.15 mmol). Flash column chromatography (1/1 EtOAc, Cyclohexane). **Yield:** 51 mg, 81 %

^1^H NMR (400 MHz, CDCl_3_, *cis*–*trans* isomers observed) δ 7.94 – 7.87 (m, 2H), 7.82 – 7.77 (m, 1H), 7.76 – 7.72 (m, 3H), 7.60 – 7.55 (m, 1H), 7.55 – 7.46 (m, 3H), 7.46 – 7.41 (m, 1H), 7.40 – 7.36 (m, 1H), 7.34 – 7.27 (m, 2H), 6.03 – 5.91 (m, 1H), 3.66 (td, *J* = 7.0, 5.9 Hz, 2H), 2.93 (t, *J* = 7.0 Hz, 2H), 2.36 (s, 3H).

^13^C NMR (101 MHz, CDCl_3_, *cis*–*trans* isomers observed) δ 167.7, 153.0, 152.7, 140.1, 139.1, 135.7, 134.8, 131.5, 131.3, 131.2, 129.8, 129.4, 129.2, 124.4, 123.0, 122.7, 121.7, 120.6, 41.1, 35.3, 21.1.

ESI-MS [C24H22N6O+H]^+^: m/z calcd. 411.19, found 411.10, t = 9.34 min, 10.43 min

HR-ESI-MS [C24H22N6O+H]^+^: m/z calcd. 411.19279, found 411.19228

**5-methyl-*N*-(4-(phenyldiazenyl)phenethyl)-2-(2*H*-1,2,3-triazol-2-yl)benzamide (2c)**

Prepared according to general procedure II from 5-methyl-2-(2*H*-1,2,3-triazol-2-yl)benzoic acid (19 mg, 0.18 mmol, 1.0 eq) and *tert*-butyl (4-(phenyldiazenyl)phenethyl)carbamate (30 mg, 0.09 mmol). Flash column chromatography (1/1 EtOAc, Cyclohexane). **Yield:** 33 mg, 87 %

^1^H NMR (400 MHz, CDCl_3_, *cis*–*trans* isomers observed) δ 7.94 – 7.88 (m, 2H), 7.88 – 7.82 (m, 2H), 7.76 (s, 2H), 7.63 – 7.58 (m, 1H), 7.55 – 7.43 (m, 3H), 7.43 – 7.38 (m, 1H), 7.36 – 7.29 (m, 3H), 5.93 (t, *J* = 5.9 Hz, 1H), 3.69 – 3.59 (m, 2H), 2.90 (t, *J* = 7.0 Hz, 2H), 2.41 (s, 3H).

^13^C NMR (101 MHz, CDCl_3_, *cis*–*trans* isomers observed) δ 167.7, 152.8, 151.6, 142.3, 139.2, 135.7, 134.9, 131.4, 131.3, 131.1, 129.8, 129.6, 129.2, 128.9, 124.6, 123.3, 122.9, 121.2, 120.5, 41.1, 35.3, 21.1.

ESI-MS [C24H22N6O+H]^+^: m/z calcd. 411.19, found 411.10, t = 9.34 min, 10.49 min

HR-ESI-MS [C24H22N6O+Na]^+^: m/z calcd. 433.17473, found 433.17632

**5-methyl-*N*-(4-(phenyldiazenyl)benzyl)-2-(2*H*-1,2,3-triazol-2-yl)benzamide (3a)**

Prepared according to general procedure II from 5-methyl-2-(2*H*-1,2,3-triazol-2-yl)benzoic acid (51 mg, 0.25 mmol, 1.0 eq) and (2-(phenyldiazenyl)phenyl)methanamine (51 mg, 0.24 mmol). Flash column chromatography (40/60 EtOAc, Cyclohexane). **Yield:** 73 mg, 76 %

^1^H NMR (500 MHz, CDCl_3_, *cis*–*trans* isomers observed) δ 7.87 – 7.83 (m, 2H), 7.74 – 7.70 (m, 1H), 7.59 – 7.56 (m, 1H), 7.55 – 7.53 (m, 3H), 7.52 – 7.44 (m, 4H), 7.44 – 7.39 (m, 2H), 7.31 – 7.27 (m, 1H), 6.37 – 6.29 (m, 1H), 5.00 (d, *J* = 6.0 Hz, 2H), 2.37 (s, 3H).

^13^C NMR (126 MHz, CDCl_3_) δ 167.2, 152.8, 150.3, 139.2, 137.0, 135.8, 135.6, 131.6, 131.5, 131.5, 131.3, 130.9, 129.9, 129.3, 128.6, 124.5, 123.2, 116.2, 40.6, 21.1.

ESI-MS [C23H22N6O+H]^+^: m/z calcd. 397.17, found 397.00, t = 9.30 min, 10.32 min

HR-ESI-MS [C23H20N6O+Na]^+^: m/z calcd. 419.15908, found 419.15972

**5-methyl-*N*-(3-(phenyldiazenyl)benzyl)-2-(2*H*-1,2,3-triazol-2-yl)benzamide (3b)**

Prepared according to general procedure II from 5-methyl-2-(2*H*-1,2,3-triazol-2-yl)benzoic acid (49 mg, 0.24 mmol, 1.0 eq) and (3-(phenyldiazenyl)phenyl)methanamine (49 mg, 0.23 mmol). Flash column chromatography (Solvent A: H_2_O + 0.1 % formic acid, Solvent B: Methanol + 0.1 % formic acid, gradient solvent A: 0-15 min 95 % → 20 %, 15-19 min 20 %, 19-20 min 20 % → 5 %, 20-23 min 5 %, retention time 16.5 min). **Yield:** 22 mg, 24 %

^1^H NMR (400 MHz, CDCl_3_, *cis*–*trans* isomers observed) δ 7.96 – 7.89 (m, 2H), 7.87 – 7.80 (m, 2H), 7.70 (s, 2H), 7.63 – 7.58 (m, 1H), 7.56 – 7.44 (m, 5H), 7.42 – 7.38 (m, 1H), 7.37 – 7.32 (m, 1H), 6.32 – 6.17 (m, 1H), 4.62 (d, *J* = 5.8 Hz, 2H), 2.44 (s, 3H).

^13^C NMR (101 MHz, CDCl_3_, *cis*–*trans* isomers observed) δ 167.6, 153.0, 152.7, 139.3, 139.1, 135.8, 134.9, 131.5, 131.3, 131.2, 130.8, 129.9, 129.5, 129.3, 128.8, 124.6, 123.0, 122.5, 122.2, 120.6, 44.0, 21.2.

ESI-MS [C23H22N6O+H]^+^: m/z calcd. 397.17, found 397.00, t = 8.99 min, 10.03 min

HR-ESI-MS [C23H20N6O+Na]^+^: m/z calcd. 419.15908, found 419.15974

**5-methyl-*N*-(4-(phenyldiazenyl)benzyl)-2-(2*H*-1,2,3-triazol-2-yl)benzamide (3c)**

Prepared according to general procedure II from 5-methyl-2-(2*H*-1,2,3-triazol-2-yl)benzoic acid (48 mg, 0.24 mmol, 1.1 eq) (4-(phenyldiazenyl)phenyl)methanamine (48 mg, 0.23 mmol). Recrystallized from ethyl acetate. **Yield:** 61 mg, 68 %

^1^H NMR (400 MHz, CDCl_3_) δ 7.95 – 7.91 (m, 2H), 7.90 – 7.85 (m, 2H), 7.74 (s, 2H), 7.65 – 7.60 (m, 1H), 7.57 – 7.45 (m, 4H), 7.45 – 7.40 (m, 2H), 7.39 – 7.33 (m, 1H), 6.25 – 6.11 (m, 1H), 4.60 (d, *J* = 5.9 Hz, 2H), 2.45 (s, 3H).

^13^C NMR (101 MHz, CDCl_3_) δ 167.5, 152.7, 152.1, 141.0, 139.3, 135.8, 134.9, 131.5, 131.2, 131.1, 129.9, 129.2, 128.8, 124.6, 123.2, 123.0, 43.9, 21.1.

ESI-MS [C23H22N6O+H]^+^: m/z calcd. 397.17, found 397.10, t = 9.18 min, 10.31 min

HR-ESI-MS [C23H20N6O+H]^+^: m/z calcd. 397.17714, found 397.17692

**(4-(5-chlorobenzo[d]oxazol-2-yl)-1,4-diazepan-1-yl)(2-((4-methoxyphenyl)diazenyl)phenyl)methanone (4)**

2-((4-methoxyphenyl)diazenyl)benzoic acid (10 mg, 0.04 mmol) was dissolved in 1 ml DCM. Ethylchloroformate (3.7 µl, 0.04 mmol, 1.0 eq.) and triethylamine (0.3 ml) were added and the mixture stirred at room temperature for 1 hour. *Tert*-butyl 4-(5-chlorobenzo[d]oxazol-2-yl)-1,4-diazepane-1-carboxylate (16.5 mg, 0.05 mmol, 1.2 eq) was dissolved in 1 ml DCM and 0.1 ml TFA. After 30 minutes at room temperature the solvent was removed *in vacuo*. The residue was redissolved in 1 ml DCM and added to the mixed anhydride solution. The mixture was stirred at room temperature for 24 hours, basified with 1 N NaOH and extracted with EtOAc. Flash column chromatography (70/30 EtOAc/Cyclohexane) afforded the product as an orange solid. Yield: 17 mg, 89 %

^1^H NMR (400 MHz, CD_2_Cl_2_, Rotamers and *cis*–*trans* isomer signals observed) δ 7.87 – 7.69 (m, 2.5H), 7.56 – 7.44 (m, 1.2H), 7.44 – 7.33 (m, 0.9H), 7.33 – 7.21 (m, 0.5H), 7.24 – 7.03 (m, 2H), 7.05 – 6.94 (m, 1H), 6.97 – 6.86 (m, 1.4H), 6.87 – 6.77 (m, 0.8H) , 6.78 – 6.73 (m, 0.3H), 6.31 – 6.10 (m, 0.1H), 4.23 – 4.06 (m, 0.4H), 4.05 – 3.55 (m, 8.4H), 3.56 – 3.41 (m, 1.4H), 3.41 – 3.20 (m, 0.8H), 2.26 – 2.06 (m, 1H), 2.01 (p, *J* = 6.1 Hz, 0.1H), 1.77 – 1.57 (m, 0.8H).

^13^C NMR (101 MHz, CD_2_Cl_2_, Rotamers and *cis*–*trans* isomer signals observed) δ 170.0 (C), 169.9 (C), 163.4 (C), 163.0 (C), 162.9 (C), 162.9 (C), 160.0 (C), 152.3 (C), 148.9 (C), 148.8 (C), 148.2 (C), 148.2 (C), 148.0 (C), 147.1 (C), 147.1 (C), 146.9 (C), 145.6 (C), 145.3 (C), 136.3 (C), 136.1 (C), 131.2 (C), 131.0 (CH), 131.0 (CH), 130.9 (CH), 130.0 (CH), 129.7 (CH), 129.5 (CH), 129.5 (CH), 129.4 (CH), 129.3 (CH), 127.5 (CH), 127.4 (CH), 127.2 (CH), 127.0 (CH), 126.9 (CH), 125.2 (CH), 125.1 (CH), 123.9 (CH), 123.8 (CH), 120.4 (CH), 120.3 (CH), 120.3 (CH), 117.8 (CH), 117.4 (CH), 116.8 (CH), 116.8 (CH), 116.3 (CH), 116.3 (CH), 116.3 (CH), 114.7 (CH), 114.6 (CH), 114.0 (CH), 109.7 (CH), 109.7 (CH), 109.6 (CH), 56.0 (CH_3_), 55.9 (CH_3_), 55.8 (CH_3_), 50.4 (CH_2_), 50.1 (CH_2_), 49.6 (CH_2_), 49.2 (CH_2_), 49.1 (CH_2_), 48.8 (CH_2_), 48.6 (CH_2_), 47.2 (CH_2_), 47.0 (CH_2_), 46.9 (CH_2_), 46.1 (CH_2_), 44.9 (CH_2_), 44.7 (CH_2_), 28.8 (CH_2_), 28.2 (CH_2_), 27.5 (CH_2_), 26.3 (CH_2_).

ESI-MS [C26H24ClN5O3+H]^+^: m/z calcd. 490.16, found 489.95, t = 10.25 min, 10.50 min

HR-ESI-MS [C26H24ClN5O3+H]^+^: m/z calcd. 490.16404, found 490.16435

**(4-(5-chlorobenzo[d]oxazol-2-yl)-1,4-diazepan-1-yl)(2-methyl-5-(2*H*-1,2,3-triazol-2-yl)phenyl)methanone (5)**

Prepared according to general procedure II from 5-methyl-2-(2*H*-1,2,3-triazol-2-yl)benzoic acid (39 mg, 0.19 mmol) and 5-chloro-2-(1,4-diazepan-1-yl)benzo[d]oxazole (47 mg, 0.24 mmol, 1.0 eq). Flash column chromatography (DCM, 5% MeOH). **Yield:** 75 mg, 92 %

Spectral data in accordance with literature reports.^[9]^

ESI-MS [C22H21ClN6O2+H]^+^: m/z calcd. 437.14, found 437.00, t = 10.14 min

HR-ESI-MS [C22H21ClN6O2+H]^+^: m/z calcd. 437.14873, found 437.14942

**Synthesis of reference compound 6**

Compound **6** was synthesized according to a modified literature procedure^[10]^.

**5-fluoro-2-(1-(tetrahydro-2*H*-pyran-2-yl)-1*H*-pyrazol-5-yl)pyridine**

2-bromo-5-fluoropyridine (517 mg, 2.84 mmol) and 1-(tetrahydro-2H-pyran-2-yl)-5-(4,4,5,5-tetramethyl-1,3,2-dioxaborolan-2-yl)-1H-pyrazole (880 mg, 3.13 mmol, 1.08 eq) were dissolved in 10 ml EtOH and 5 ml toluene. The reaction mixture was stirred at room temperature overnight, after which Pd(PPh_3_)_4_ and 4 ml 2 M K_2_CO_3_ were added. The mixture was stirred at 90 °C for 7 hours, water (30 ml) was added and the aqueous phase extracted with EtOAc. Flash column chromatography (30/70 EtOAc/Cyclohexane) afforded the product as a colorless oil. **Yield**: 650 mg, 90 %

^1^H NMR (400 MHz, CDCl_3_) δ 8.54 (d, *J* = 2.9 Hz, 1H), 7.65 – 7.58 (m, 2H), 7.47 (td, *J* = 8.4, 2.9 Hz, 1H), 6.54 (d, *J* = 1.9 Hz, 1H), 6.10 (dd, *J* = 10.0, 2.5 Hz, 1H), 4.08 – 3.97 (m, 1H), 3.63 – 3.53 (m, 1H), 2.61 – 2.45 (m, 1H), 2.16 – 1.97 (m, 2H), 1.77 – 1.62 (m, 2H), 1.59 – 1.49 (m, 1H).

^13^C NMR (101 MHz, CDCl_3_) δ 160.1, 157.5, 146.2, 146.2, 141.1, 139.4, 137.9, 137.6, 124.7, 124.7, 123.9, 123.7, 107.6, 85.0, 68.0, 29.8, 25.1, 23.1.

ESI-MS [C13H14FN3O-THP+H]^+^: m/z calcd. 164.05, found 164.15, [C13H14FN3O+Na]^+^: m/z calcd. 270.10, found 269.90, t = 8.844 min

**5-Fluoro-2-(1*H*-pyrazol-3-yl)pyridine**

5-fluoro-2-(1-(tetrahydro-2*H*-pyran-2-yl)-1*H*-pyrazol-5-yl)pyridine (550 mg, 2.22 mmol) was dissolved in 1 ml methanol and 1.2 ml 4 M HCl/EtOAc was added. The mixture was stirred over night, after which 0.5 ml TFA was added and the mixture stirred for an additional 3 days at room temperature. To this was added 10 ml H_2_O, the mixture was adjusted to pH 13 with 1 N NaOH and extracted with EtOAc. Flash column chromatography (70/30 EtOAc/Cyclohexane +1% Et_3_N) afforded the product as a colorless solid. **Yield**: 188 mg, 52 %

^1^H NMR (500 MHz, CDCl_3_) δ 11.70 (s, 1H), 8.51 (d, *J* = 2.9 Hz, 1H), 7.80 (s, 1H), 7.66 (d, *J* = 2.2 Hz, 1H), 7.50 – 7.42 (m, 1H), 6.78 (s, 1H).

^13^C NMR (126 MHz, CDCl_3_) δ 160.0, 158.0, 137.8, 137.7, 124.0, 123.9, 121.3, 121.2, 103.5.

ESI-MS [C8H6FN3+H]^+^: m/z calcd. 164.05, found 164.15, t = 7.442 min

***tert*-Butyl (2-[3-(5-fluoropyridin-2-yl)-1*H*-pyrazol-1-yl]ethyl)carbamate**

5-Fluoro-2-(1*H*-pyrazol-3-yl)pyridine (20 mg, 0.12 mmol), *tert*-butyl-*N*-(2-bromoethyl)carbamate (36.5 mg, 0.16 mmol, 1.3 eq) and Cs_2_CO_3_ (63 mg, 0.19 mmol, 1.6 eq) were dissolved in 0.5 ml DMF and the mixture was stirred at 80 °C for 7 hours. Water (30 ml) was added and the aqueous phase extracted with EtOAc. Flash column chromatography (1/1 EtOAc/Cyclohexane) afforded the product as a colorless solid. **Yield**: 21 mg, 56 %

^1^H NMR (500 MHz, CDCl_3_) δ 8.46 (d, *J* = 2.9 Hz, 1H), 7.92 – 7.89 (m, 1H), 7.44 – 7.39 (m, 2H), 6.80 (d, *J* = 2.3 Hz, 1H), 4.86 (s, 1H), 4.30 – 4.24 (m, 2H), 3.63 – 3.58 (m, 2H), 1.42 (s, 9H).

^13^C NMR (126 MHz, CDCl_3_) δ 159.9, 157.9, 156.0, 151.6, 148.8, 148.7, 137.6, 137.5, 131.8, 123.6, 123.5, 121.1, 106.8, 104.3, 79.8, 61.4, 51.9, 43.5, 40.9, 28.5, 28.1.

ESI-MS [C15H19FN4O2+H]^+^: m/z calcd. 307.15, found 307.30, t = 9.119 min

***N*-(2-(3-(5-fluoropyridin-2-yl)-1*H*-pyrazol-1-yl)ethyl)-5-methyl-2-(2*H*-1,2,3-triazol-2-yl)benzamide (6)**

*tert*-Butyl (2-[3-(5-fluoropyridin-2-yl)-1*H*-pyrazol-1-yl]ethyl)carbamate (19.5 mg, 63 µmol) was dissolved in 1 ml DCM and 0.1 ml TFA and stirred at room temperature for 4 hours. The solvent was removed *in vacuo*, 5-methyl-2-(2H-1,2,3-triazol-2-yl)benzoic acid (16.5 mg, 81 µmol, 1.3 eq) HBTU (32 mg, 84 µmol, 1.3 eq), DMF (1 ml) and DIPEA (0.1 ml) were added and the mixture was stirred at room temperature overnight. The reaction mixture was diluted with H_2_O and extracted with EtOAc. Flash column chromatography (80/20 EtOAc/Cyclohexane) afforded the product as a colorless solid. **Yield**: 19 mg, 76 %

^1^H NMR (500 MHz, Acetone, two sets of signals observed) δ 8.46 (d, *J* = 2.9 Hz, 1H), 8.08 – 8.03 (m, 1H), 7.91 (s, 2H), 7.81 – 7.79 (m, 1H), 7.68 – 7.65 (m, 1H), 7.65 – 7.58 (m, 2H), 7.42 – 7.39 (m, 1H), 7.38 – 7.36 (m, 1H), 6.80 (d, *J* = 2.3 Hz, 1H), 4.39 (dd, *J* = 6.5, 5.5 Hz, 2H), 3.80 – 3.74 (m, 2H), 2.37 (s, 3H).

^13^C NMR (126 MHz, Acetone, two sets of signals observed) δ 168.2, 167.8, 160.7, 160.6, 158.7, 158.6, 151.9, 150.3, 150.3, 147.2, 139.2, 139.2, 138.0, 137.8, 137.8, 137.6, 136.5, 136.4, 136.1, 132.9, 132.6, 131.6, 131.5, 130.2, 130.2, 125.4, 125.4, 125.2, 125.1, 124.6, 124.5, 124.2, 124.1, 121.5, 121.4, 107.5, 104.7, 51.8, 50.5, 41.0, 40.9, 20.9.

ESI-MS [C20H18FN7O+H]^+^: m/z calcd. 392.16, found 392.35, t = 8.832 min

HR-ESI-MS [C20H18FN7O+H]^+^: m/z calcd. 392.16296, found 392.16357

# **NMR and LC-MS Characterization**

## **LC-MS Data of Target Compounds**

**Figure S41:** HPLC-MS-chromatograms of photoswitchable orexin ligand **1a**

**Figure S42:** HPLC-MS-chromatograms of photoswitchable orexin ligand **1b**

**Figure S43:** HPLC-MS-chromatograms of photoswitchable orexin ligand **1c**

**Figure S44:** HPLC-MS-chromatograms of photoswitchable orexin ligand **2a**

**Figure S45:** HPLC-MS-chromatograms of photoswitchable orexin ligand **2b**

**Figure S46:** HPLC-MS-chromatograms of photoswitchable orexin ligand **2c**

**Figure S47:** HPLC-MS-chromatograms of photoswitchable orexin ligand **3a**

**Figure S48:** HPLC-MS-chromatograms of photoswitchable orexin ligand **3b**

**Figure S49:** HPLC-MS-chromatograms of photoswitchable orexin ligand **3c**

**Figure S50:** HPLC-MS-chromatograms of photoswitchable orexin ligand **4**

**Figure S51:** HPLC-MS-chromatograms of reference orexin ligand **5**

**Figure S52:** HPLC-MS-chromatograms of reference orexin ligand **6**

## **NMR Spectra**

# **Computational Methods and Results**

## **Docking Protocol**

Receptor structures (**Figure S54**) were prepared using the Maestro Protein Preparation Workflow (version 14.3.129, MMshare Version 6.9.129, Release 2025-1, Platform Linux-x86_64)^[11]^. The conserved water molecule mediating a hydrogen bond with His7x39 was retained. Missing hydrogen atoms were added, automatically optimizing the hydrogen bond network (**Figure S55**). Structures were refined by restrained energy minimization of the entire system (receptor, ligand, and conserved water) using the Optimized Potentials for Liquid Simulations (OPLS) 4 force field. Sampling of water orientations was activated. Heavy atoms were restrained to a maximum RMSD of 0.3 Å.

Ligands were protonated in MOE v2022.02^[12]^ by washing at pH 7.4 to generate the most probable protomer in both *cis*- and *trans*-states. For each prepared isomer, 30 conformers were generated with RDKit v2023.03.3 (AllChem.EmbedMultipleConfs) and geometry-optimized using the Universal Force Field (UFFOptimizeMoleculeConfs). The generation of conformers was found necessary to capture the flexibility of the diazepane ring of suvorexant.

Conformer ensembles were docked using Smina (Oct 15 2019 build). The docking box was centered at (42, 40, –53) with dimensions 20 × 20 × 20 Å, covering the orthosteric binding site. The top-scoring pose across all conformers was selected as the representative binding mode of the ligand (**Figures S56** and **S58**–**60**).

The prepared receptors, ligands, and docking parameters, as well as all docking outputs, are available at https://github.com/vicenledesma/TClock4AD_DC4/tree/main/Photorexant.

## **Alkylene Linker Length Analysis**

To investigate the replacement of the diazepane ring, variants of the docked ligands *cis*-v27, *trans*-v27, and *trans*-v29 in which the diazepane ring was substituted by alkylene linkers of length *n* = 0–3 were generated using RDKit. For each variant, 10,000 conformers were generated as described in the docking protocol. Atom mapping was performed between shared substructures of the alkylene-linked derivatives and the docked reference ligands. The substructures that were matched were: the methylphenyl-triazole with the amide and the azobenzene (SMARTS-based matching using Chem.rdchem.Mol.GetSubstructMatches). The conformers were then rigidly aligned to the template docking pose on the matched substructures using Chem.rdMolAlign, and RMSD values were calculated for said substructures. The conformer exhibiting the lowest RMSD was retained for further analysis (results in **Figure S58**). The script used for rigid alignment of molecules based on defined shared substructures, as well as the results of the alkyl linker length analysis, can be found at https://github.com/vicenledesma/TClock4AD_DC4/tree/main/Photorexant.

**Figure S53:** The 26 photoswitchable suvorexant derivatives generated by the computational pipeline after applying the molecular weight filter (300–600 Da).

**Figure S54:** Superposition of the crystallographic structures of the orexin receptors in complex with suvorexant (left), and close-up view (right) of the binding poses of suvorexant in OX1R (blue) and OX2R (orange). The structures correspond to PDB IDs 6TO7 and 4S0V, respectively. Red dashed lines indicate a direct hydrogen bond to N318/324^6×55^ and a water-mediated hydrogen bond to H344/350^7×39^.

**Figure S55:** Receptor refinement (protonation and energy minimization) for docking induces the flip of residue Q134^3×32^ in OX2R to maximize hydrogen bonding network (red dashed lines). In the OX2R refined structure, the Y354^7x43^ hydroxy-H forms a hydrogen bond with T111^2x61^, while the T111^2x61^ hydroxy-H is engaged in a hydrogen bond with the backbone carbonyl oxygen of C107^2x57^. In turn, this favors an orientation of the Q134^3×32^ side chain with the amide nitrogen directed toward the Y354^7x43^ hydroxy-O, potentially influencing ligand docking poses. To address this in the docking studies, an OX2R structure with the reverted orientation of Q134^3×32^ was prepared.

**Figure S56:** Summary of docking results of the 26 photoswitchable suvorexant derivatives to the three prepared orexin receptor structures (OX1R, OX2R, and OX2R Q134^3×32^ flipped). **A**. Docking scores in *cis*- vs *trans*-conformation for the docked ligands. **B**. Root mean square deviation (RMSD) values of the docked ligands to the co-crystallized suvorexant, calculated over maximum common induced subgraph (MCIS) atoms. The lowest value corresponds to the self-docking RMSD of suvorexant (docking validation), followed by the *cis*- and *trans-* photoswitch MCIS RMSDs, sorted by 2D structural similarity (Tanimoto similarity over Morgan fingerprints, 2048 bits) to suvorexant (annotated on the right side of the plot as Tanimoto coefficient, *Tc*).

**Figure S57:** Ligand alignments for alkylene linker length analysis. Shown are the best alignments between the docked ligands, used as templates, and the alkylene-linked variants. The first column shows the virtual screening hits superimposed with suvorexant bound to OX1R (PDB ID: 6TO7). Columns two through five show the virtual screening hits aligned with alkylene-linked variants in which the diazepane ring was replaced by alkylene linkers of length *n* = 0–3. Root-mean-square deviation values for the indicated matched substructures (RMSD_sub_) between the diazepane (docked) and the alkyl-linked photoswitchable derivatives (aligned) are indicated below each alignment.

**Figure S58:** Docking poses of compounds *cis*-**2b** and *cis*-**3b** in OX2R. The surface of co-crystallized suvorexant (transparent) is also displayed. Despite experimental results suggesting a preference for a longer ethyl-linker, docking predictions show similar orientations, approaching the horseshoe-shaped binding mode of suvorexant, but with a rotated methylphenyltriazole moiety.

**Figure S59:** Docking poses of *para*-substituted azobenzene compounds from sets 2 and 3 (**2c** and **3c**) in OX1R and OX2R, showing a horseshoe binding mode even though they display the weakest antagonistic activity experimentally.

**Figure S60:** Structural comparison of ligand binding in OX1R. **A**. Crystal structures of OX1R bound to urea-containing antagonists SB-334867 (PDB ID: 6TQ7) and SB-408124 (PDB ID: 6TQ9). These structures display two copies of each ligand occupying the binding site and extending into the subpocket between transmembrane helices 5 (TM5) and TM6. The urea-linked moiety that extends is highlighted in yellow. **B**. Predicted docking pose of photorexant (**4**) suggesting that the azobenzene and the additional methoxy group (both highlighted in blue) are accommodated in the same subpocket.

**SI Figure S61:** Predicted binding modes of *cis*- and *trans*-photorexant (**4**) docked into OX1R and OX2R, compared to the experimental binding mode of suvorexant in OX1R (PDB ID: 6TO7) and OX2R (PDB ID: 4S0V). Maximum common induced subgraph (MCIS) RMSD values relative to co-crystallized suvorexant are reported, showing good agreement (<2 Å) for the *trans*-photoisomer across all structures, whereas the *cis*-photoisomer appears displaced. Transmembrane domains are labeled with Roman numerals. *Trans*-photoisomers are colored green and *cis*-photoisomers purple.

# **References**

[1]	A. Cannaert, J. Storme, F. Franz, V. Auwärter, C. P. Stove, “Detection and activity profiling of synthetic cannabinoids and their metabolites with a newly developed bioassay” *Anal. Chem.* **2016**, *88*, 11476–11485.

[2]	E. Pottie, D. K. Tosh, Z.-G. Gao, K. A. Jacobson, C. P. Stove, “Assessment of biased agonism at the A3 adenosine receptor using β-arrestin and miniGαi recruitment assays” *Biochem. Pharmacol.* **2020**, *177*, 113934.

[3]	E. Pottie, P. Dedecker, C. P. Stove, “Identification of psychedelic new psychoactive substances (NPS) showing biased agonism at the 5-HT2AR through simultaneous use of β-arrestin 2 and miniGαq bioassays” *Biochem. Pharmacol.* **2020**, *182*, 114251.

[4]	M. H. Deventer, M. Angermann, S. Mori, M. Decker, C. P. Stove, “Sleep, wake, and signaling: Functional profiling of orexin agonists and antagonists using newly developed orexin β-arrestin 2 and miniGαq recruitment assays” *Eur. J. Pharmacol.* **2026**, *(accepted)*.

[5]	A. Cannaert, L. Vasudevan, M. Friscia, A. L. A. Mohr, S. M. R. Wille, C. P. Stove, “Activity-Based Concept to Screen Biological Matrices for Opiates and (Synthetic) Opioids” *Clin. Chem.* **2018**, *64*, 1221–1229.

[6]	E. Schaller, S. Lamer, A. Schlosser, C. Stigloher, P. Maher, M. Decker, “Affinity-Based Protein Profiling Reveals IDH2 as a Mitochondrial Target of Cannabinol in Receptor-Independent Neuroprotection” *Chem. Eur. J.* **2025**, *31*, e202501143.

[7]	G. Paxinos, K. B. J. Franklin, *The mouse brain in stereotaxic coordinates*, Elsevier Academic Press, Amsterdam Heidelberg, **2004**.

[8]	E. J. Craven, J. Latham, S. A. Shepherd, I. Khan, A. Diaz-Rodriguez, M. F. Greaney, J. Micklefield, “Programmable late-stage C−H bond functionalization enabled by integration of enzymes with chemocatalysis” *Nat. Catal.* **2021**, *4*, 385–394.

[9]	J. Hellmann, M. Drabek, J. Yin, J. Gunera, T. Pröll, F. Kraus, C. J. Langmead, H. Hübner, D. Weikert, P. Kolb, D. M. Rosenbaum, P. Gmeiner, “Structure-based development of a subtype-selective orexin 1 receptor antagonist” *Proc. Natl. Acad. Sci. U. S. A.* **2020**, *117*, 18059–18067.

[10]	R. Suzuki, D. Nozawa, A. Futamura, R. Nishikawa-Shimono, M. Abe, N. Hattori, H. Ohta, Y. Araki, D. Kambe, M. Ohmichi, S. Tokura, T. Aoki, N. Ohtake, H. Kawamoto, “Discovery and in vitro and in vivo profiles of N-ethyl-N-[2-[3-(5-fluoro-2-pyridinyl)-1H-pyrazol-1-yl]ethyl]-2-(2H-1,2,3-triazol-2-yl)-benzamide as a novel class of dual orexin receptor antagonist” *Bioorg. Med. Chem.* **2015**, *23*, 1260–1275.

[11]	Schrödinger Release 2025-1: Protein Preparation Workflow; Epik, Schrödinger, LLC, New York, NY, 2024; Impact, Schrödinger, LLC, New York, NY; Prime, Schrödinger, LLC, New York, NY **2025**.

[12]	Molecular Operating Environment (MOE), 2022.02 Chemical Computing Group ULC, 910-1010 Sherbrooke St. W., Montreal, QC H3A 2R7 **2022**.
